# SPIKE-1: A Randomised Phase II/III trial in a community setting, assessing use of camostat in reducing the clinical progression of COVID-19 by blocking SARS-CoV-2 Spike protein-initiated membrane fusion

**DOI:** 10.1186/s13063-021-05461-9

**Published:** 2021-08-19

**Authors:** Sarah Halford, Susan Wan, Ilaria Dragoni, Julie Silvester, Bobojon Nazarov, Daniel Anthony, Suzie Anthony, Emma Ladds, John Norrie, Kevin Dhaliwal, the CDD SPIKE-1 Project Team

**Affiliations:** 1 Cancer Research UK Centre for Drug Development, London, UK; 2 Latus Therapeutics, London, UK; 3 University of Oxford, Oxford, UK; 4 Oxford University Hospitals NHS Foundation Trust, Oxford, UK; 5 Nuffield Department of Primary Care Health Sciences, University of Oxford, Oxford, UK; 6 The University of Edinburgh, Edinburgh, UK

**Keywords:** COVID-19, Randomised controlled trial, protocol, camostat, TMPRSS2, Spike

## Abstract

**Objectives:**

The primary objective is to evaluate the efficacy of camostat to prevent respiratory deterioration in patients with Severe Acute Respiratory Syndrome Coronavirus 2 (SARS-CoV-2) infection.

Secondary objectives include assessment of the ability of camostat to reduce the requirement for Coronavirus disease 2019 (COVID-19) related hospital admission and to reduce the requirement for supplementary oxygen and ventilation as treatment for SARS-CoV-2 infection, to evaluate overall mortality related to COVID-19 and to evaluate the efficacy of camostat by effect on clinical improvement.

Research objectives include to assess change in COVID-19 symptom severity, to evaluate the ability of camostat to reduce viral load throughout duration of illness as well as translational research on host and viral genomics, serum antibody production, COVID-19 diagnostics, and validation of laboratory testing methods and biomarkers.

**Trial design:**

SPIKE-1 is a randomised, multicentre, prospective, open label, community-based clinical trial. Eligible patients will be randomised 1:1 to the camostat treatment arm and control arm (best supportive care). The trial is designed to include a pilot phase recruiting up to 50 patients in each arm. An initial review at the end of the pilot phase will allow assessment of available data and inform the requirement for any protocol adaptations to include refinement of eligibility criteria to enrich the patient population and sample size calculations. Up to 289 additional patients will be randomised in the continuation phase of the trial. A formal interim analysis will be performed once 50% of the maximum sample size has been recruited

**Participants:**

The trial will recruit adults (≥ 18 years) who score moderate to very high risk according to COVID-age risk calculation, with typical symptoms of COVID-19 infection as per Public Health England guidance or equivalent organisations in the UK, Health Protection Scotland, Public Health Wales, Public Health Agency (Northern Ireland) and with evidence of current COVID-19 infection from a validated assay.

The trial is being conducted in the UK and patients are recruited through primary care and hospital settings.

**Intervention and comparator:**

Eligible patients with be randomised to receive either camostat tablets, 200 mg four times daily (qds) for 14 days (treatment arm) or best supportive care (control arm).

**Main outcomes:**

Primary outcome measure: the rate of hospital admissions requiring supplemental oxygen.

Secondary outcome measures include: the rate of COVID-19 related hospital admission in patients with SARS-CoV-2 infection; the number of supplementary oxygen-free days and ventilator-free days measured at 28 days from randomisation; the rate of mortality related to COVID-19 one year from randomisation; the time to worst point on the nine-point category ordinal scale (recommended by the World Health Organization: Coronavirus disease (COVID-2019)) or deterioration of two points or more, within 28 days from randomisation.

Research outcomes include the assessment of change in COVID-19 symptom severity on days 1-14 as measured by (1) time to apyrexia (maintained for 48 hrs) by daily self-assessment of temperature, time to improvement (by two points) in peripheral oxygenation saturation defined by daily self-assessment of fingertip peripheral oxygenation saturation levels, (3) assessment of COVID-19 symptoms using the Flu-iiQ questionnaire (determined by app recording and/or daily video call (or phone) consultation and (4) assessment of functional score (where possible) at screening, day 7 and 14. The ability of camostat to reduce viral load throughout duration of illness will be assessed by (1) change in respiratory (oropharyngeal/nasopharyngeal swab RT-PCR) log10 viral load from baseline to Days 7 and 14, (2) change in respiratory (saliva RT-PCR) log10 viral load from baseline to Days 1-14 and (3) change in upper respiratory viral shedding at Day 1 -14 measured as time to clearance of nasal SARS-CoV-2, defined as 2 consecutive negative swabs by qPCR.

Additional translational research outcomes include assessment of host and viral genomics, serum antibody production and COVID-19 diagnostics at baseline and on Days 7 and 14.

**Randomisation:**

Eligible patients will be randomised using an interactive web response system (IWRS) in a 1:1 ratio to one of two arms: (1) treatment arm or (2) control arm.

**Blinding (masking):**

The trial is open-label.

**Numbers to be randomised (sample size):**

The trial is designed to include a pilot and a continuation phase. Up to 100 patients (randomised 1:1 treatment and control arm) will be recruited in the pilot phase and a maximum of 289 patients (randomised 1:1 treatment and control) will be recruited as part of the continuation phase. The total number of patients recruited will not exceed 389.

**Trial Status:**

Protocol version number v3 25 September 2020. Trial opened to recruitment on 04 August 2020. The authors anticipate recruitment to be completed by October 2021.

**Trial registration:**

EudraCT 2020-002110-41; 18 June 2020

ClinicalTrials.gov NCT04455815; 02 July 2020

**Full protocol:**

The full protocol is attached as an additional file, accessible from the Trials website (Additional file 1). Unpublished PK data provided under confidentiality agreement to the trial Sponsor has been removed from the background section of the protocol to allow for publication of the trial protocol. In the interest in expediting dissemination of this material, the familiar formatting has been eliminated; this Letter serves as a summary of the key elements of the full protocol.

**Supplementary Information:**

The online version contains supplementary material available at 10.1186/s13063-021-05461-9.

## Supplementary Information

**Additional file 1.** Full study protocol.

## Data Availability

On completion of the study, results will be published on ClinicalTrials.gov. Additionally, following approval by the funder, Ono Pharmaceutical Co. Ltd. and Sponsor, full data will be made available to academic researchers upon reasonable request.

