# Supplementary material for: SPIKE-1: A Randomised Phase II/III trial in a community setting, assessing use of camostat in reducing the clinical progression of COVID-19 by blocking SARS-CoV-2 Spike protein-initiated membrane fusion
Source: Trials. 2021 Aug 19;22:550. doi: 10.1186/s13063-021-05461-9 (PMC8375281; doi:10.1186/s13063-021-05461-9)
Supplement: Supplementary file 1 [file 13063_2021_5461_MOESM1_ESM.docx]

**SPIKE-1 TRIAL:** A Randomised Phase II/III trial in a community setting, assessing use of camostat in reducing the clinical progression of 

COVID-19 by blocking SARS-CoV-2 Spike protein-initiated membrane fusion.

**Date and Version No**: 	25th September 2020, Version 3.0 **(for publication)**

**Protocol No.:**			CRUKD/20/002

**EudraCT No.:**			2020-002110-41

             

**Chief Investigator:**		Professor Kevin Dhaliwal, University of Edinburgh

**Sponsor:**			Cancer Research UK

Centre for Drug Development

2 Redman Place

London E20 1JQ

**Co-Investigators:**		Prof Bruce Guthrie, The University of Edinburgh

Prof Brian McInstry, The University of Edinburgh 

Dr Ahsan Akram, The University of Edinburgh 

Dr Keith Finlayson, The University of Edinburgh

Dr Annya Bruce, The University of Edinburgh

Prof John Norrie, The University of Edinburgh

Prof Daniel Anthony, University of Oxford 

Dr Colin Ferrett, Oxford University Hospitals NHS Foundation Trust

				Prof Andrew Beggs, University of Birmingham

 				Dr Grant Churchill, University of Oxford

Dr Bobojon (Islom) Nazarov, University of Oxford & Latus Therapeutics

Dr Suzie Anthony, Oxford University Hospitals NHS Foundation Trust

				Dr Emma Ladds, University of Oxford, Primary Care

	

**PARTICIPATING INVESTIGATORS AND CENTRES:**

Details of Chief Investigator (CI), Principal Investigators (PIs) and investigational sites are recorded on the Participating Investigators and Centres list in the Sponsor’s Trial Master File (TMF).

**Confidentiality Statement****:**

This document contains confidential information that must not be disclosed to anyone other than the Sponsor, the Investigator Team, host NHS Trust/Boards(s), regulatory authorities, and members of the Research Ethics Committee (REC).  

**SPONSOR STATEMENT:**

This project is supported by Cancer Research UK (CR UK) in support of the response to the coronavirus-19 (COVID-19) pandemic.  CR UK researchers and collaborators across the globe turned their expertise and infrastructure towards COVID-19 research and this project is supported and sponsored by CR UK as part of that global effort. The full extent of the disruption COVID-19 will have on cancer services and people affected by cancer is unprecedented therefore CR UK supports COVID-19 research with the ambition of ensuring cancer prevention, screening and treatment services, are restored and available to those affected by cancer.

**AMENDMENT HISTORY:**

| **Protocol Version No. and Date** | **Summary of changes** |
| --- | --- |
| 1.0 dated 26MAY2020 | Initial version for REC/HRA and MHRA submission |
| 2.0 dated 04JUN2020 | Post initial submission changes to include: Table1: Typo correction, pregnancy test added. Section 5.4.2.1: New section, ‘Dose interruption/Withdrawal criteria.’ Appendix 2: FluiiQ update for COVID-19 symptoms and administrative updates. Section 6.2: Exclusion criteria #2 added related to Liver Function Test result. |
| 3.0 dated 25SEP2020 | **Substantial** Section 6.1: Inclusion criteria and reference to COVID-age for participant eligibility (associated Appendix 4 for COVID-age risk tool) Section 6.1: Reference to COVID-19 symptoms will be as per guidance issued by the UK health authorities. Section 7.2.5: Camostat administration to be taken in a fasted state (rationale provided in revised Section 3.0) **Non-substantial** Amendment history table: Liver Function Test exclusion criteria added (omitted in error from v2.0 summary of changes). Other minor updates/clarifications. |

TABLE OF CONTENTS

1	ABBREVIATIONS	8

2	SYNOPSIS	12

3	Background and Rationale	16

3.1	Investigational Medicinal Product – Camostat	17

3.2	Proposed dose and dosing schedule for CRUKD/20/002	17

3.3	Safety considerations	18

3.3.1	Adverse events	18

3.3.2	Patient population	18

3.3.3	Risk and benefit assessment	18

4	OBJECTIVES and endpoints	19

5	TRIAL DESIGN	21

5.1	Summary of trial design	21

5.2	Safety oversight and committees	23

5.2.1	Trial Management Group	23

5.2.2	Trial Steering Committee	23

5.2.3	Independent Data Monitoring Committee	23

5.2.4	Safety oversight	24

5.3	Interim reviews	24

5.3.1	Pilot stage	24

5.3.2	Interim analysis	24

5.4	Intervention	24

5.4.1	Randomisation	24

5.4.2	Dose modification	25

5.4.2.1	Dose interruptions/ Withdrawal criteria	25

5.4.3	Day 7 visit	25

5.4.4	Patient hospital admission during Days 1-14	26

5.4.5	Additional pharmacokinetic sampling	26

5.4.6	Concomitant medications	26

5.4.7	Trial partners	26

5.4.8	Expenses and benefits	26

6	patient selection	27

6.1	Inclusion Criteria	27

6.2	Exclusion Criteria	27

7	pharmaceutical INFORMATION	29

7.1	Supply of Investigational Medicinal Product	29

7.2	Pharmaceutical Data	29

7.2.1	Formulation of camostat	29

7.2.2	Storage conditions	29

7.2.3	Stability and labelling of camostat	29

7.2.4	Dispensing of camostat	30

7.2.5	Camostat administration	30

7.2.6	Camostat accountability	30

7.2.7	Camostat supply to primary and secondary care pharmacies	30

8	Trial procedures	31

8.1	Baseline assessments	31

8.2	Confirmation of eligibility	32

8.3	On study assessments/procedures	32

8.3.1	Daily for video call (or phone) monitoring: Days 1-14	32

8.3.2	Home visit consultation with clinical professional: Days 7 and 14	32

8.3.3	Video call (or phone) monitoring: Days 21 and 28	33

8.3.4	Days 1-28	33

8.3.5	Long term follow up	33

8.4	Sample collection and transport	34

8.5	Escalation of patient management	34

9	Recruitment	37

9.1	Identification of patients	37

9.1.1	Testing at COVID hubs (GP practices or walk-in centres)	37

9.1.2	Other settings	38

9.2	Informed Consent	38

10	Definition of End of Trial	39

11	Discontinuation/ Withdrawal of PaTIENTs from Study Treatment	40

12	SAFETY REPORTING	41

12.1	Definitions	41

12.1.1	Adverse Event (AE)	41

12.1.2	Adverse Drug Reaction (ADR)	41

12.1.3	Serious Adverse Event (SAE)	41

12.1.4	Suspected Unexpected Serious Adverse Reaction (SUSAR)	42

12.1.5	Urgent Safety Measures (USM)	42

12.2	Causality	42

12.3	Procedures for Recording Adverse Events	43

12.4	Procedures for recording pregnancies	43

12.5	Reporting Procedures for Serious Adverse Events	44

12.6	Specific Events of Interest	44

12.7	Annual Development Safety Update Reports (DSUR)	45

13	data analysis and statistical considerations	46

13.1	Description of statistical methods	46

13.2	The Level of Statistical Significance	47

13.3	Sample size determination	47

13.4	Subgroup analysis	47

13.5	Interim reporting	47

13.6	Clinical Study Report	47

14	DATA MANAGEMENT	48

14.1	Source data	48

14.2	Access to data	48

14.3	Data recording and record keeping	48

14.3.1	Recording of daily calls	48

15	QUALITY CONTROL AND QUALITY ASSURANCE PROCEDURES	49

15.1	Risk assessment	49

15.2	Monitoring	49

15.3	Quality assurance	49

15.4	Protocol deviations and amendments	50

15.5	Serious Breach of GCP	50

16	ETHICal considerations	50

16.1	Approvals	50

16.2	Patient confidentiality	51

16.3	Indemnity	51

17	References	52

18	appendices	53

18.1	Appendix 1: The Chelsea Critical Care Physical Assessment tool (Cpax) – Copyright of Chelsea and Westminster NHS Foundation Trust (01MAR2010) (Corner, 2012)	53

18.2	Appendix 2: COVID-19-symptom collection using the Influenza Intensity and Impact Questionnaire (FluiiQ™) - provided free of charge by Measured Solutions for Health and tailored for COVID-19 trials.	54

18.3	Appendix 3: New York Heart Association (NYHA) scale	56

18.4	Appendix 4: COVID-age calculation	57

# 1 ABBREVIATIONS 

| A&E | Accident and Emergency |
| --- | --- |
| ABPI | Association of British Pharmaceutical Industry |
| ACEI | Angiotensinogen-converting enzyme inhibitors |
| ACE-2 | Angiotensin-converting enzyme 2 |
| ADR | Adverse drug reaction |
| AE | Adverse event |
| ALAMA | Association of Local Authority Medical Advisors |
| ALP | Alkaline phosphatase |
| ALT | Alanine aminotransferase |
| APTT | Activated partial thromboplastin time |
| AR | Adverse reaction |
| AST | Aspartate aminotransferase |
| BNP | B-type Natriuretic Peptide |
| CDD | Centre for Drug Development |
| CDM | Clinical Data Manager |
| CI | Chief Investigator |
| C_max_ | Maximum observed plasma concentration |
| CoA | Certificate of Analysis |
| COVID-19 | Coronavirus disease 2019 |
| Cpax | Chelsea Critical Care Physical Assessment tool |
| CRA | Clinical Research Associate |
| CRP | C-reactive protein |
| CRUK | Cancer Research UK |
| CSM | Clinical Study Manager |
| CSR | Clinical Study Report |
| CT | Computerised tomography |
| CTA | Clinical Trials Authorisation |
| CTIMP | Clinical Trial of an Investigational Medicinal Product |
| CTRG | Clinical Trials & Research Governance, University of Oxford |
| CXR | Chest X-ray |
| DMP | Data Management Plan |
| DSUR | Development Safety Update Report |
| EC50 | Half maximal effective concentration |
| ECMO | Extracorporeal membrane oxygenation |
| eCRF | Electronic Case Report Form |
| EoT | End of Trial |
| FBC | Full blood count |
| FluiiQ | Influenza Intensity and Impact Questionnaire |
| GBPA | 4-(4-guanidinobenzoyloxy) phenyl acetic acid |
| GGT | Gamma-glutamyl transferase |
| GCP | Good Clinical Practice |
| GDPR | General Data Protection Regulation |
| GMP | Good Manufacturing Practice |
| GP | General Practitioner |
| GTP | Gamma-glutamyl transpeptidase |
| Hb | Haemoglobin |
| HRA | Health Research Authority |
| IB | Investigator’s Brochure |
| IC_10_ | 10% of maximal inhibitory concentration |
| IC_50_ | Half maximal inhibitory concentration |
| ICD | Informed Consent Document |
| ICH | International Conference of Harmonisation |
| ID | Identification |
| iDMC | Independent Data Monitoring Committee |
| IMP | Investigational Medicinal Product |
| IV | Intravenous |
| IWRS | Interactive web response system |
| LFT | Liver function tests |
| MERS-CoV | Middle East Respiratory Syndrome coronavirus |
| MHRA | Medicines and Healthcare products Regulatory Agency |
| NLR | Neutrophil:lymphocyte ratio |
| NHS | National Health Service |
| NSAIDs | Non-steroidal anti-inflammatory drugs |
| NYHA | New York Heart Association |
| PI | Principal Investigator |
| PK | Pharmacokinetic |
| PSRB | Protocol and Safety Review Board |
| PT | Prothrombin time |
| qds | *quater die sumendum* (to be taken four times daily) |
| QP | Qualified Person |
| qPCR | Quantitative (real-time) polymerase chain reaction |
| REC | Research Ethics Committee |
| RNA | Ribonucleic acid |
| RRT | Renal replacement therapy |
| RT-PCR | Reverse transcription polymerase chain reaction |
| SAE | Serious Adverse Event |
| SAP | Statistical Analysis Plan |
| SAR | Serious Adverse Reaction |
| SARS-CoV | Severe Acute Respiratory Syndrome coronavirus |
| SARS-CoV-2 | Severe Acute Respiratory Syndrome coronavirus 2 |
| SmPC | Summary of Product Characteristics |
| SUSAR | Suspected Unexpected Serious Adverse Reaction |
| tds | *ter die sumendum* (to be taken three times daily) |
| T_1/2_ | Terminal half-life |
| T_max_ | Time to maximum concentration |
| TMF | Trial Master File |
| TMG | Trial Management Group |
| TSC | Trial Steering Committee |
| UK | United Kingdom |
| U&E | Urea and electrolytes |
| USM | Urgent Safety Measure |
| Vd | Volume of distribution |
| WBC | White Blood Cells |

**PROTOCOL SIGNATURES**

**Sponsor Signature**

The Sponsor has read and agrees to the protocol, as detailed in this document. I am aware of my responsibilities as the Sponsor under the UK Clinical Trials Regulations[^1], the guidelines of Good Clinical Practice (GCP)[^2], the Declaration of Helsinki[^3], the applicable regulations of UK law and the trial protocol. The Sponsor agrees to conduct the trial according to these regulations and guidelines and to appropriately direct and assist sponsor’s staff who will be involved in the trial and ensure that all staff members are aware of their clinical trial responsibilities.

| Name: |  |  |
| --- | --- | --- |
|  |  |  |
| Title |  |  |
|  |  |  |
| Signature: |  |  |
|  |  |  |
| Date: |  |  |

**PROTOCOL SIGNATURES**

**Chief Investigator Signature**

I have read and agree to the protocol, as detailed in this document. I am aware of my responsibilities as a Chief Investigator under the UK Clinical Trials Regulations^1^, the guidelines of Good Clinical Practice (GCP)^2^, the Declaration of Helsinki^3^, the applicable regulations of the relevant NHS Trust/Boards and the trial protocol. I agree to conduct the trial according to these regulations and guidelines and to appropriately direct and assist the staff under my control, who will be involved in the trial, and ensure that all staff members are aware of their clinical trial responsibilities.

| Chief Investigator's Name: |  |  |
| --- | --- | --- |
|  |  |  |
| Name of site: |  |  |
|  |  |  |
| Signature: |  |  |
|  |  |  |
| Date: |  |  |

[^4]

# 2 SYNOPSIS

| **Study Title** | A Randomised Phase II/III trial in a community setting, assessing use of camostat in reducing the clinical progression of COVID-19 by blocking SARS-CoV-2 Spike protein-initiated membrane fusion. |
| --- | --- |
| **Short title** | SPIKE-1 |
| **Sponsor** | Cancer Research UK |
| **Funder** | Latus Therapeutics (via LifeArc grant award) |
| **Trial Design** | A randomised, multicentre, prospective, open label, community-based clinical trial. There are two arms: **Treatment arm:** Patient to receive treatment with camostat tablets, 200mg four times daily (*qds*) for 14 days **Control arm (non-treatment):** Patient to receive best supportive care. The trial will be split into two parts: **Pilot phase:** This phase will randomise eligible patients between two arms; the treatment arm and the control arm. Patients will be randomised 1:1 into each of these arms until both arms have recruited up to 50 patients. Data from the pilot will undergo continuous review, potentially allowing an earlier decision before 50 patients have been recruited in each of the arms. A pilot review will take place once both arms have recruited up to 50 patients in each arm. This interim review will allow assessment of the pilot phase data and allow for adaptation of the continuation phase to include, trial feasibility, refinement of eligibility criteria to enrich the patient population and sample size calculations. **Continuation phase:** This phase will allow further investigation of camostat by continuing to randomise patients into the treatment arm or the control arm. Early assessment of recruitment and trial feasibility will be critical therefore the Trial Management Group (TMG) will provide ongoing review of these aspects and propose modification during the pilot and continuation phase for regulatory/ethical approval as appropriate. |
| **Trial patients** | Adults, 18 years of age and above who score moderate to very high risk according to COVID-age risk calculation. With typical symptoms of COVID-19 infection as per Public Health England guidance or equivalent organisations in the UK, Health Protection Scotland, Public Health Wales, Public Health Agency (Northern Ireland). Evidence of current COVID-19 infection from a validated assay Patients will be recruited from testing centres which may include primary care ‘COVID-19 hub’ clinics, COVID-19 community-based testing centres or equivalent clinical environments. |
| **Sample size** | **Pilot phase:** Up to 100 patients (randomised 1:1 treatment and control arm). **Continuation phase (including formal interim analysis at 50%):** maximum 289 patients (randomised 1:1 treatment and control). Total patients recruited (maximum): 389 patients |
| **Planned trial period** | The trial will start as soon as permissions are in place and procedures and structures implemented. The anticipated end date for this trial is November 2021 however that may be amended based on emerging data and circumstances. |
| **Planned recruitment period** | **Pilot phase:** Approximately 6 months **Continuation phase:** Approximately 8 months |
| **Primary Objective and Endpoint** |  |
| **Objective** | **Endpoint** |
| To evaluate the efficacy of camostat to prevent respiratory deterioration in patients with Severe Acute Respiratory Syndrome Coronavirus 2 (SARS-CoV-2) infection. | Hospital admission requiring supplemental oxygen. |
| **Secondary Objectives and Endpoints** |  |
| To assess the ability of camostat to reduce the requirement for COVID-19 related hospital admission in patients with SARS-CoV-2 infection. | Rate of COVID-19 related hospital admission in patients with SARS-CoV-2 infection. |
| To evaluate the requirement for supplementary oxygen (non-invasive or mechanical invasive) in patients who have received camostat as treatment for SARS-CoV-2 infection. | Supplementary oxygen-free days at 28 days (from randomisation). |
| To evaluate the requirement for ventilation in patients who have received camostat as treatment for SARS-CoV-2 infection. | Ventilator-free days at 28 days (from randomisation). |
| To evaluate overall mortality. | Mortality related to COVID-19, one year from randomisation. |
| To evaluate efficacy of camostat by effect on clinical improvement. | Time Frame: Days 1-28 Time to worst point on the scale or deterioration of two points or more (from randomisation) on a 9-point category ordinal scale. 9-point category ordinal scale: 0. Uninfected, no clinical or virological evidence of infection 1. Ambulatory, no limitation of activities 2. Ambulatory, limitation of activities 3. Hospitalised – mild disease, no oxygen therapy 4. Hospitalised – mild disease, oxygen by mask or nasal prongs 5. Hospitalised – severe disease, non-invasive ventilation or high-flow oxygen 6. Hospitalised – severe disease, intubation and mechanical ventilation 7. Hospitalised – severe disease, ventilation and additional organ support e.g. vasopressors, renal replacement therapy (RRT), extracorporeal membrane oxygenation (ECMO) 8. Death |
| **Research Objectives and Endpoints** |  |
| To assess change in COVID-19 symptom severity. | Time Frame: Days 1-14 Time to apyrexia (maintained for 48 hrs) as defined by daily self-assessment of temperature (digital). Time to improvement (by two points) in peripheral oxygenation saturation defined by daily self-assessment of fingertip peripheral oxygenation saturation levels. Assessment of COVID-19 symptoms using the Flu-iiQ questionnaire (determined by app recording and/or daily video call (or phone) consultation.  Assessment of functional score where possible (Screening, Days 7 and 14). |
| To evaluate the ability of camostat to reduce viral load throughout duration of illness by oropharyngeal/nasopharyngeal and/or saliva swab RT-PCR. | Change in respiratory (oropharyngeal/nasopharyngeal swab RT-PCR) log10 viral load from baseline to Days 7 and 14.  Change in respiratory (saliva RT-PCR) log10 viral load from baseline to Days 1-14 (if performed). Change in upper respiratory viral shedding at Day 1 -14. Time to clearance of nasal SARS-CoV-2, defined as 2 consecutive negative swabs by qPCR. |
| Translational research on host and viral genomics, serum antibody production, COVID-19 diagnostics, and validation of laboratory testing methods and biomarkers. | Time Frame: Baseline, Days 7 and 14 Analysis of research samples collected at baseline prior to treatment and at specific time points. Biochemical/haematological parameters measured in venous blood at Baseline, Days 7 and 14 days and venous sample real-time PCR on Days 7 and 14. |
| **Safety and tolerability** |  |
| Camostat is a drug with 30 years of clinical experience in Japan and South Korea where it is in clinical use for treatment of an unrelated condition (chronic pancreatitis) and postoperative reflux oesophagitis, with a known, acceptable safety profile. Daily self-assessment of peripheral oxygen saturation and temperature (digital) with daily video call (or phone) consultation of all patients will support early reporting of study compliance. Patients will be given clear information regarding peripheral oxygen saturation thresholds, indications of progressive breathlessness, or other concerning symptoms that should trigger immediate self-referral to seek health advice from the appropriate channels as they normally would, according to urgency e.g. General Practitioner (GP) or dialling National Health Service (NHS) 111 or 999. Daily video call (or phone) assessments will support reinforcement and safety netting of these features, allowing for self- or clinical trial team referral for appropriate primary care, 111 or 999 review, in accordance with local clinical pathways. |  |
| **Trial assessments** |  |
| **Screening (Home visit):** Demographics, medical history, height and weight. Haematological and biochemical parameters measured in blood.  Research blood samples taken for other markers and antibody testing (if available). Fingertip peripheral oxygen saturation and pulse rate. Temperature (digital). Oropharyngeal/nasopharyngeal swabs (or other validated method) for COVID-19 (if not previously tested). Saliva (viral load) test (if available), for batch analysis. Number of patients asked to perform this test and days of testing may be adapted based on test availability. COVID-19 symptom collection using the Flu-iiQ questionnaire (see Appendix 2.0). Collection of symptoms and functional score. **All patients (all arms):** **Days 1-14** Daily video call (or phone) monitoring for all patients. COVID-19 symptom collection using the Flu-iiQ questionnaire (see Appendix 2.0). Collection of symptoms. Daily self-assessment of fingertip peripheral oxygen saturation and pulse rate. Daily self-assessment of temperature (digital). Daily measurement of saliva (viral load) test – if available, for batch analysis. Number of patients asked to do these tests and days of testing may be adapted based on test availability.  **Days 7 (+/- 1 day) and 14 (- 2days)** Home visit and consultation with clinical professional. The intention is that home visits occur on Days 7 and 14 to take research bloods and repeat swab.  However, this requirement may change if patients are able to provide samples by visiting the hospital (or other suitable clinical site) but only in accordance with government and clinical advice.  If the Sponsor and site decide that sampling is not required, this will be communicated to the patient.  The Day 7 and 14 video (phone) calls will still go ahead as planned regardless of sampling. Oropharyngeal/nasopharyngeal swabs (or other validated method) for COVID-19 viral load. Haematological and biochemical parameters measured in blood.  Research blood samples taken for other markers and antibody testing (if available). Collection of functional score (where possible). **Days 21 and 28** Video call (or phone) monitoring for all patients. COVID-19 symptom collection using the Flu-iiQ questionnaire (see Appendix 2.0). Collection of symptoms. Self-assessment of fingertip peripheral oxygen saturation and pulse rate. Self-assessment of temperature (digital). **Days 1-28** Review of hospital and primary care records if applicable to evaluate use of any form of supplementary oxygen, imaging (chest X-ray or computerised tomography [CXR/CT scanning]) CXR/CT reports or images to assess progression or evolution of pneumonia and evidence of pulmonary thromboembolic events plus documentation of death (COVID related mortality) during the trial period. Patients who are hospitalised will be followed up following discharge. At Day 28 post discharge, the patient will be contacted to collect information on existing/new symptoms and also any additional results from COVID-19 testing. **Long term follow-up:** Survival at 1-year post randomisation. |  |

# 3 Background and Rationale

There is currently a critical lack of approved therapies for COVID-19, caused by the severe acute respiratory syndrome coronavirus-2 (SARS-CoV-2) virus (Liu, 2020) and the main standard of care is supportive treatment only (Arabi, 2020) (Ruan, 2019). Recently, remdesivir received conditional marketing authorisation in the EU (03 July 2020) for the treatment of COVID 19 in adults and adolescents with pneumonia requiring supplemental oxygen. There is also an ongoing evaluation by the European Medicines Agency of a marketing authorisation for dexamethasone for the treatment of hospitalised adult patients with COVID 19. The clinical manifestations of COVID-19 range from asymptomatic infections or mild, transient symptoms to severe viral pneumonia with respiratory failure. Although many patients do not progress to severe disease, a significant number are hospitalised with pneumonia as the SARS-CoV-2 infection spreads.

SARS-CoV-2 is an enveloped, positive-sense, single-stranded RNA β-coronavirus similar to the severe acute respiratory syndrome (SARS) and Middle East respiratory syndrome (MERS) coronaviruses. Cell entry of coronaviruses, such as SARS-CoV and MERS-CoV, depend on initial binding of the viral spike (S) proteins to the angiotensin-converting enzyme 2 (ACE-2) receptor, found on the cell surface of target cells and subsequent priming of the S protein by host cell proteases. The serine protease type II transmembrane serine protease (TMPRSS2), also expressed at the cell surface, ‘primes’ the S protein on the virus facilitating its activation and enabling the fusion of the virus and target cell membranes and transfer of viral RNA (Shulla, 2011) (Shirato, 2013); this process is shown schematically in Figure 1. Inhibition of TMPRSS2 in *in vitro* and *in vivo* models has been shown to play a significant role in reducing viral infection and viral spread (Matsuyama, 2010) (Shulla, 2011) (Iwata-Yoshikawa, 2019). 

SARS-CoV-2 has been shown to also infect human cells via binding of its spike (S) protein to the ACE-2 receptor on the cell surface and priming of the S protein by the cellular serine protease, TMPRSS2, which facilitates its activation and enables the fusion of the virus with the host cell (Yan, 2020).

Figure 1 Schematic of TMPRSS2/blocking of SARS-CoV-2 Spike protein-initiated membrane fusion

(A) Spike proteins on the surface of the coronavirus bind to angiotensin-converting enzyme 2 (ACE-2) receptors on the surface of the host cell membrane; (B) the type II transmembrane serine protease (TMPRSS2) binds to and cleaves the ACE-2 receptor. In the process the spike protein is activated; (C) Cleaved ACE-2 and activated spike protein facilitate viral entry. TMPRSS expression increases cellular uptake of the coronavirus.

Source: (Rabi, 2020).

Camostat has been shown to inhibit the serine protease TMPRSS2 and block SARS-CoV (Kawase, 2012) and SARS-CoV-2 (Hoffmann, 2020) (Hoffmann, APR 2020) infection of human epithelial lung cells *in vitro* and improved survival outcomes in a mouse model of SARS-CoV (Zhou, 2015). Camostat significantly reduced SARS-CoV-2 viral entry in Calu-3 cells with an EC_50_ of 87 nM and no interference in cell viability reported (Hoffmann, APR 2020). This reported EC_50_ is consistent with IC_50_’s determined in other cell-based assays investigating the inhibition of SARS-CoV and MERS-CoV viral entry by camostat, which have been in the range of 1 to 0.1 µM (Hoffmann, SARS-CoV-2 Cell Entry Depends on ACE2 and TMPRSS2 and is Blocked by a Clinically Proven Protease Inhibitor, 2020) (Kawase, 2012) and (Yamamoto, 2016).

Camostat may therefore present a potential solution to treat COVID-19 infected patients.

For further information on the background and rationale for investigating camostat as a potential treatment for COVID-19 refer to the current version of the ‘Camostat Investigator’s Brochure’.

## Investigational Medicinal Product – Camostat

Camostat is a non-peptide serine protease inhibitor and after oral administration, acts promptly on kinin formation, fibrinolytic, coagulation and complementary systems to inhibit enzyme activities and their abnormal increases. It has also been shown to inhibit TMPRSS2, a host cell factor involved in the cellular entry of coronaviruses (Kawase, 2012) (Hoffmann, SARS-CoV-2 Cell Entry Depends on ACE2 and TMPRSS2 and is Blocked by a Clinically Proven Protease Inhibitor, 2020) (Hoffmann, APR 2020).

Camostat is rapidly hydrolysed to its active metabolite 4-(4-guanidinobenzoyloxy) phenyl acetic acid (GBPA) *in vivo*; the inhibitory effects of both camostat and GBPA on serine proteases have been shown to be comparable *in vitro*.

Camostat (camostat mesilate, FOIPAN®) is licensed for clinical use in Japan and South Korea and was first approved for use in these countries in 1985 and 1989, respectively. It is marketed by Ono Pharmaceutical Co., Ltd. and has been in clinical use for over 30 years. Camostat is indicated for the treatment of acute symptoms of chronic pancreatitis (recommended dose 600 mg [200 mg *tds*]; guidance on administration in a fed or fasted state is not given) and for the treatment of postoperative reflux oesophagitis (recommended dose 300 mg [100 mg *tds*]; doses to be taken after food); it has a known, acceptable safety profile. Camostat is not approved for use in the EU for any indication and will be used outside of its licensed indication and investigated for the treatment of COVID-19 in the CRUKD/20/002 trial.

For further information on camostat, refer to the current version of the ‘Camostat Investigator’s Brochure’.

## Proposed dose and dosing schedule for CRUKD/20/002

***NOTE: unpublished PK data has been removed from this section (Protocol Version 3.0, dated 25 Sept 2020) to allow for publication of the CRUKD/20/002 protocol.  The data removed cannot be disclosed under the terms of contractual agreements in place between the Sponsor (Cancer Research UK) and Ono Pharmaceutical Co., Ltd.***

The concentration of camostat required to suppress viral entry and significantly affect SARS-CoV, MERS-CoV or SARS-CoV-2 infection has not been determined rigorously *in vivo* but can be informed from the *in vitro* EC_50_ of 0.087 µM (Hoffmann, APR 2020) and estimated IC_50_’s in the range 1 to 0.1 µM (Hoffmann, 2020) (Kawase, 2012) and (Yamamoto, 2016). 

An important consideration in determining the recommended dose and schedule for this trial is whether the concentration of GBPA (active metabolite of camostat[^5]) achieved after dosing in humans, is sufficient to achieve plasma concentrations in this range (target concentration considered to be 0.1 µM). The reported PK parameters for a 200 mg oral dose of camostat (2 x 100 mg tablets) administered in a fasted state are: C_max_ 87 ng/mL; T_max_ 40 min; AUC 10,400 ng.min/mL; T_1/2_ 100 min. The C_max_ corresponds to a concentration of 0.18 µM. This is within the range of camostat concentrations that have been shown to inhibit viral entry *in vitro*.  

On the basis of this PK data, dosing camostat in a fasted state  at 200 mg every 8 hours (the approved dosing schedule), and given a T_1/2_ of 100 min, the plasma concentration of camostat would only be expected to remain above the IC_50_ of 0.1 µM for approximately 2 hours after each dose.  

Options to increase exposure levels and the duration of exposure above the target level of 0.1 µM in the CRUKD/20/002 trial, were considered by the Sponsor and a total daily dose of 800 mg (200 mg *qds*), for 14 days was chosen for this trial.

Camostat has been in clinical use for over 30 years and is indicated for the treatment of acute symptoms of chronic pancreatitis (recommended dose 600 mg [200 mg tds]) and for the treatment of postoperative reflux oesophagitis (recommended dose 300 mg [100 mg tds]). Dosing instructions in the approved Package Insert state doses should be taken after a meal for the treatment of postoperative reflux oesophagitis but no guidance on dosing in a fed or fasted state is given for the treatment of chronic pancreatitis. 

The proposed camostat daily dose and schedule for use in the CRUKD/20/002 trial is not, however, approved for use in clinical practice.

## Safety considerations

For further information on the safety of camostat and specific safety considerations refer to the current version of the ‘Camostat Investigator’s Brochure’.

### 3.1.1 Adverse events

Camostat has been in clinical use for over 30 years in Japan and Korea and has a known, acceptable safety profile. Adverse reactions reported during the safety surveillance of camostat for its licensed indications include shock or anaphylactoid symptoms, thrombocytopenia, changes in liver enzymes. gastrointestinal disorders, rash and hyperkalaemia, all with low reported incidences (<1% patients).

### 3.1.2 Patient population

The majority of clinical studies supporting the original registration of FOIPAN® in Japan were conducted in a Japanese/Asian population. 

Two Phase I PK studies have been performed in Caucasians, one by Forrest,1989 and one by Midgley, 1994. Camostat is rapidly metabolised in vivo by a carboxyesterase to its active metabolite GBPA and the inactive by product 4-guanidinobenzoic acid (GBA) (Midgley, 1994). The active metabolite GBPA is further metabolized by arylesterase, glucuronidated and excreted in the urine (Midgley, 1994). Genetic variations due to ethnicity have not been reported for any of these enzymes involved in the metabolism of camostat. From the available data, it is concluded that the PK/PD differences between the Asian and Caucasian populations are predicted to be minimal and that the known safety of camostat in Asians and Caucasians is expected to be similar (Forrest, 1989) (Midgley, 1994).

### 3.1.3 Risk and benefit assessment

For the overall risk and benefit assessment for the investigation of camostat in protocol CRUKD/20/002, refer to Section 5.8 in the current version of the ‘Camostat Investigator’s Brochure’.

# 4 OBJECTIVES and endpoints

| **Primary Objective and Endpoint** |  |
| --- | --- |
| **Objective** | **Endpoint** |
| To evaluate the efficacy of camostat to prevent respiratory deterioration in patients with SARS-CoV-2 infection. | Hospital admission requiring supplemental oxygen. |
| **Secondary Objectives and Endpoints** |  |
| **Objective** | **Endpoint** |
| To assess the ability of camostat to reduce the requirement for COVID-19 related hospital admission in patients with SARS-CoV-2 infection. | Rate of COVID-19 related hospital admission in patients with SARS-CoV-2 infection. |
| To evaluate the requirement for supplementary oxygen (non-invasive or mechanical invasive) in patients who have received camostat as treatment for SARS-CoV-2 infection. | Supplementary oxygen-free days at 28 days (from randomisation). |
| To evaluate the requirement for ventilation in patients who have received camostat as treatment for SARS-CoV-2 infection. | Ventilator-free days at 28 days (from randomisation). |
| To evaluate overall mortality. | Mortality related to COVID-19, one year from randomisation. |
| To evaluate efficacy of camostat by effect on clinical improvement. | Time Frame: Days 1-28 Time to worst point on the scale or deterioration of two points or more (from randomisation) on a 9-point category ordinal scale. 9-point category ordinal scale: 0. Uninfected, no clinical or virological evidence of infection 1. Ambulatory, no limitation of activities 2. Ambulatory, limitation of activities 3. Hospitalised – mild disease, no oxygen therapy 4. Hospitalised – mild disease, oxygen by mask or nasal prongs 5. Hospitalised – severe disease, non-invasive ventilation or high-flow oxygen 6. Hospitalised – severe disease, intubation and mechanical ventilation 7. Hospitalised – severe disease, ventilation and additional organ support – vasopressors, renal replacement therapy (RRT), extracorporeal membrane oxygenation (ECMO) 8. Death |
| **Research Objectives and Endpoints** |  |
| **Objective** | **Endpoint** |
| To assess change in COVID-19 symptom severity. | Time Frame: Days 1-14 Time to apyrexia (maintained for 48 hrs) as defined by daily self-assessment of temperature (digital). Time to improvement (by two points) in peripheral oxygenation saturation defined by daily self-assessment of fingertip peripheral oxygenation saturation levels. Assessment of COVID-19 symptoms using the Flu-iiQ questionnaire (determined by app recording and/or daily video call (or phone) consultation. Assessment of functional score (where possible)  (Screening, Days 7 and 14). |
| To evaluate the ability of camostat to reduce viral load throughout duration of illness by oropharyngeal/nasopharyngeal and/or saliva swab RT-PCR. | Change in respiratory (oropharyngeal/nasopharyngeal swab RT-PCR) log10 viral load from baseline to Days 7 and 14. Change in respiratory (saliva RT-PCR) log10 viral load from baseline to Days 1-14 (if performed). Change in upper respiratory viral shedding at Day 1 -14. Time to clearance of nasal SARS-CoV-2, defined as 2 consecutive negative swabs by qPCR. |
| Translational research on host and viral genomics, serum antibody production, COVID-19 diagnostics, and validation of laboratory testing methods and biomarkers. | Time Frame: Baseline, Days 7 and 14 Analysis of research samples collected at baseline prior to treatment and at specific time points. Biochemical/haematological parameters measured in venous blood at Baseline, Days 7 and 14 days and venous sample real-time PCR on Days 7 and 14. |

# 5 TRIAL DESIGN

## Summary of trial design

A randomised, multicentre, prospective, open label, community-based clinical trial. 

**Figure 4 Trial design**

**Patient identified**

**and provides consent**

**Agreement with TMG and TSC to continue to randomise patients to continuation phase**

**Patient randomised**

**Interim review to assess pilot data and adapt trial design as appropriate**

**Treatment arm**

**camostat**

**200mg,** ***qds*** **for 14 days**

**up to 50 pts**

**Control arm**

**Best Supportive Care**

**up to 50 pts**

**Throughout trial:**

**Ongoing safety review by Sponsor, TMG and TSC**

**Please see Section** **9** **for further details regarding patient identification and consent.**

**Treatment arm** 

**camostat**

**200mg,** ***qds*** **for 14 days**

**up to 145pts**

**Control arm**

**Best Supportive Care**

**up to 145 pts**

**Pilot phase:**

**Adaptive design with data reviewed throughout to support endpoints and trial feasibility**

**Continuation phase:** 

**To include formal interim analysis by the iDMC**

TMG: Trial Management Group

TSC: Trial Steering Committee 

iDMC: Independent Data Monitoring Committee

The trial has been designed as a pilot study, followed by a continuation phase.

The TMG described in Section 5.2.1, will perform ongoing review of the emerging data to inform adaptive decision making during the pilot and in support of further adaptations required for the continuation phase. Please see Section 5.3.1. for further details. The pilot phase will focus on trial and recruitment feasibility. 

During both the pilot and continuation phase, patient enrichment strategies may be implemented *e.g.* adapting the inclusion criteria to include emerging co-morbidities, neutrophil/lymphocyte ratio, other blood biomarkers, age, in order to support trial endpoints and feasibility. All clinical data will be reviewed on a regular basis by the Sponsor and TMG to inform this adaptive pilot phase, collaborating with the Trial Steering Committee (TSC) as per Section 5.2.2. 

A review of the pilot phase will be performed when up to 50 patients have been recruited into each of the two arms. A decision can then be made regarding whether the trial should progress into the continuation phase and what further adaptations are required.  This review will include clinical data, and possible re-estimation of the sample size, based on a review of the aggregated (blinded) event rate for the primary outcome and a check on the rate of missing primary outcomes (Section 5.3.1).  Further proposals may also be made to enrich the patient population. A formal interim analysis will be performed by the independent Data Monitoring Committee (iDMC) (Section 5.2.3) when approximately 50% of the maximum sample size have been recruited, Section 5.3.2.

If review of the data (during any part of the trial) results in proposed substantial amendments, these will be submitted to the Medicines and Healthcare products Regulatory Agency (MHRA) and Research Ethics Committee (REC) for an opinion.  

Patients may present/be identified in various settings e.g. primary care COVID hubs/community-based testing centre, drive through testing facilities or equivalent clinical settings. Further detail on how patients will be recruited is provided in Section 9.  It is likely that testing availability and strategy will change in the UK and the trial will respond by engaging with testing sites as applicable, to support recruitment. Before any new sites are opened for this trial, risk assessments will be performed to ensure that the protocol and procedures can be performed safely for both patients and site staff.

If a patient consents to the trial and fulfils eligibility criteria, they will be randomised to either the treatment arm or the control arm. Patients in the treatment arm will be asked to take camostat for 14 consecutive days and patients in the control arm will be asked to continue with best supportive care. Both groups will be asked to take daily temperature, pulse rate and peripheral oxygen saturation readings for 14 days. A clinical research team member will call the patient daily during this period. On Days 7 and 14, patients will be visited at home by the clinical research team, for blood draws and COVID-19 swab (or equivalent) testing.  Home visits may be replaced by patients visiting a designated hub/GP practice/testing centre but only in accordance with government and clinical advice. If the Sponsor and site decide that sampling is not required, this will be communicated to the patient.  The video (phone) calls on Days 7 and 14 will still go ahead as planned regardless of sampling. Patients will also be called weekly (Days 21 and 28) before the follow up period ends.  

Adverse events (AEs) or hospital admissions related to camostat or COVID-19 will be followed up as per Section 12.0.

During the trial period (Days 1-28), patients who are hospitalised will also be followed up, 28 days after they are discharged. The patient will be contacted to collect information on existing/new symptoms and also any additional results from COVID-19 testing.

Patients will be asked to consent to follow up for one-year post randomisation, to collect recovery or survival data via hospital and primary care records, GP or by contacting the patient.  

For 90% power at a 5% level of significance, on the primary outcome of hospitalisation requiring supplemental oxygen (yes or no), assuming that in the enriched cohort the control rate will be 40%, and the in the camostat group that will reduce by an absolute 16% (or a relative reduction of 40%) to 24%, the study will require 177/group or 354 in total. Adjusting this sample size for 1 interim analysis at 50% with mature primary outcome data using a group sequential design (Hwang-Shih-DeCani spending function with O’Brien Fleming bounds) increases this sample size to 370. Assuming that 5% will be lost to follow up on the primary outcome, the trial is required to recruit 370/0.95 = 389 participants. Therefore, the trial aims to recruit 100 in the pilot phase and up to a maximum of 289 in the continuation phase. 

## Safety oversight and committees 

### 5.1.1 Trial Management Group

The Trial Management Group is responsible for the day to day running of the trial including but not limited to, the Sponsor, CI, Trial Statistician, PIs and Co-Investigators who will have oversight of all clinical and safety data throughout both phases of the trial. A core study team will oversee management of the trial and make decisions at the operational level. At the pilot review, between the pilot and continuation phase, the Trial Steering Committee (TSC) (Section 5.2.2), convened by the Sponsor, will review the data and proposed amendments, alongside the TMG.  

Early assessment of recruitment and trial feasibility will be critical therefore the TMG will provide ongoing review of emerging clinical and feasibility data during the pilot phase and propose modification where required for regulatory/ethical approval as appropriate. The TSC will be asked for an opinion as appropriate when these modifications are proposed by the TMG.

### 5.1.2 Trial Steering Committee

### A TSC will be convened to review safety and efficacy data from the trial on an ongoing basis.  The TSC will include the CI, Sponsor representatives including the Medical Advisor, at least two independent experts with expertise in COVID infection and/or other relevant specialities, trial statistician and one member of the Sponsor’s Protocol and Safety Review Board[^6] (PSRB).

The TSC will provide expert advice in the event of any concerns around toxicity and will review safety and efficacy data prior to the continuation phase and during the pilot phase as requested by the TMG. The TSC will have the authority to suspend or terminate the trial and/or make recommendations to the Sponsor to modify the trial design.

Meetings of the TSC will be convened by the Clinical Study Manager (CSM) for the trial to review data generated at time points where key decisions are required. Additional ad hoc meetings may be convened should unforeseen events occur, and independent expert advice would be beneficial.

The responsibility for calling and organising TSC meetings lies with the CSM for the trial in association with the Chair of the TSC. Meetings will take place at least every six months if a review has not already taken place. The TSC, CI or Sponsor can request additional meetings at any time.

A TSC Charter will be made available to support the trial. The charter will include reference to the Statistical Analysis Plan (SAP) and how results from any analysis will be communicated to the TSC to inform decision making.

### 5.1.3 Independent Data Monitoring Committee

An iDMC will be convened to perform the formal interim analysis (when 50% of the maximum sample size has been recruited). The iDMC will consist of a Chair, an additional clinician with knowledge of the safety issues, and an independent statistician. 

### 5.1.4 Safety oversight

Safety evaluations by the TMG will be conducted at regular intervals throughout patient recruitment and at the interim review. Data reviewed includes all clinical data, safety data listings and trial feasibility. Any concerns relating to safety can be escalated to the TSC for an opinion and recommendation.

## Interim reviews

### 5.1.5 Pilot stage 

The first review will take place at the end of the pilot phase, when both arms have recruited up to 50 patients who have mature data on the primary outcome of hospitalisation requiring supplemental oxygen. This review will consider many aspects of study performance, including the efficiency of recruitment (how many potential participants need to be approached to establish eligibility; and how many of the established to be eligible were willing to be randomised); compliance with study procedures (how many complied with the protocol); in the camostat arm, compliance with randomised medication; and the rate of missing data for the primary outcome (assumed to be 5%). These study performance metrics will be jointly reviewed to take a decision on whether the study can continue unchanged or needs some modifying (e.g. more centres and/or more time to recruit; or improved interaction with the patients), or whether continuing to the full trial is not feasible, in which case the study would stop. In addition, at the end of this pilot stage, there will be a review of the aggregated (blinded) event rate. It is assumed that in the control group the event rate is 40% and in the treated arm 24%. Therefore, across the two arms 32% is expected, and so for 100 pilot patients, it is expected that 32 events will be observed, with a 95% confidence interval of 23 to 41 events. Therefore, if the aggregate number of events is between 23 and 41, the trial will continue with the original sample size of up to 389 (assuming 5% loss to follow up). If the observed events are 22 or less, or 42 or more, the trial will re-estimate the sample size after discussions of what an appropriate absolute and relative percentage treatment effect would be, given the lower or higher overall event rate. All of these discussions on the study performance and aggregated event rate only require blinded information, and so will be mediated by the Trial Steering Committee.  

### 5.1.6 Interim analysis

Assuming the study progresses into the continuation phase (the full trial) we will include a formal interim analysis to allow the study to terminate either because of overwhelming evidence of benefit, or because it is futile to continue (the observed treatment  at the time of the time of the interim analysis was sufficiently small that it would take an unfeasibly strong turnaround of events in the remaining part of the trial yet unobserved to make the overall treatment effect statistically significant and clinically worth having). This will take place when approximately 50% of the maximum sample size have been recruited and achieved primary outcome status (up to 28 days after randomisation). This interim analysis will use a group sequential design with a Hwang-Shih-DeCani spending function with O’Brien and Fleming boundary which is non-binding. This formal interim analysis will consider unblinded information about the emerging treatment effect and will therefore be conducted in strict confidence by the Independent Data Monitoring Committee and the unblinded study statistician.   

## Intervention

### 5.1.7 Randomisation

Randomisation will occur in both the pilot phase and continuation phase. Patients will be randomised by the site staff using an interactive web response system (IWRS). Confirmation of the patient randomisation will be sent to the Investigator, site clinical trial team and Sponsor following enrolment and randomisation of the patient. The patient will be notified of their allocated group by the clinical research team.

In the pilot phase, randomisation will be to one of two arms. A randomisation ratio of 1:1 will be applied:

- **Treatment arm:** patient to receive treatment with camostat 200 mg *qds* for 14 days. 

- **Control arm:** patients will receive best supportive care.

In the continuation phase, randomisation will be to one of two arms. A randomisation ratio of 1:1 will be applied:

- **Treatment arm:** patient to receive treatment with camostat 200 mg *qds* for 14 days. 

- **Control arm:** patients will receive best supportive care.

All patients randomised will receive a trial pack to include a digital thermometer and fingertip peripheral oxygen saturation probe, both with instructions for use as well as a diary card to record these readings and, if randomised to camostat, to document their compliance with dosing. The research team or courier will deliver the appropriate trial pack.

Patients randomised to the treatment arm will also be offered best supportive care in managing the disease.

### 5.1.8 Dose modification

The planned dose for all patients who are randomised to the treatment arm is 800 mg per day (200 mg *qds*) for 14 days. Dose modifications may occur such as reducing the daily dose. Dose reductions for individual patients may be considered based on emerging clinical data and if agreed with the Sponsor, CI and PIs.  These dose reductions could take the form of reduction of times that camostat is administered e.g. *qds* to *tds* or to a reduction at each of the daily administrations e.g. 200 mg *qds*, down to 100 mg *qds*. Based on any interim review that takes place during the pilot phase, a change to the dose for all patients going forward, may also be considered.

The patient will be contacted daily during the treatment period by the clinical research team for assessment of any AEs and difficulties the patient may be experiencing with the administration schedule. 

Escalation of patient management is further discussed in Section 8.5.

#### 5.1.8.1 Dose interruptions/ Withdrawal criteria

- If a clinically significant abnormality is identified, camostat will be interrupted and  blood samples will be taken for analysis. 

- If abnormal results are identified, the patient will be referred to the GP for further assessment where local protocols will be followed.

- If required, patient hospital attendance with a suspected adverse event will trigger SAE reporting and the Investigator and Sponsor will make a causality assessment.

- Possible, Probably and Highly Probable causality assessment to camostat will result in treatment withdrawal.

- If the patient recovers symptomatically and biochemical analysis returns to the normal range, re-starting camostat can be considered by the Investigator and Sponsor to include an assessment whether the patient is likely to be past the initial stage of infection. For patients where it is deemed appropriate to re-start the patient, a maximum of 48 hrs treatment interruption will be allowable.

### 5.1.9 Day 7 visit

It is intended that the Day 7 interaction with the patient is made by a clinical professional who can assess whether the patient has recovered. Patients may be visited at home on Day 7 (to collect research bloods and swab (and saliva samples if taken)) as well as receiving the daily video (phone) call.  If at this visit/during the call, it is assessed that the patient (on the treatment arm) has recovered, the patient may be asked to discontinue with camostat administration, and the drug will be disposed of as per an approved process. Follow up calls and visits as per protocol will continue. 

### 5.1.10 Patient hospital admission during Days 1-14

If during Days 1-14 the patient is admitted to hospital, patients on the treatment arm will be asked to continue to take their camostat as per protocol following agreement from the treating clinician. A patient identification (ID) card will be provided to all patients with relevant contact details. Patients requiring hospital admission will continue to take camostat unless contraindicated by their clinical condition or other clinical care.

For patients who are hospitalised, clinical research teams should still seek to perform the protocol mandated assessments as per Section 8.3, so long as they do not interfere with clinical care and management of the patient.

### 5.1.11 Additional pharmacokinetic sampling

The Sponsor may propose that for patients who are hospitalised, if they have consented, and so long as the sampling schedule does not interfere with their hospital care, PK blood samples may be taken at Sponsor defined timepoints for analysis. 

The patient will be asked to consent to this when first recruited to the trial and then asked again for verbal affirmation of consent (recorded in the patient’s medical record) before these blood samples are taken.  If the patient is unable to give consent or incapacitated, these samples will not be taken.

A maximum of 4 mL of blood will be taken at each timepoint for this analysis.

During the course of the trial, patients who remain able to recover at home, may also be asked to consent to PK sampling.  Patients will be asked to consent to this at the time of recruitment and verbal affirmation of consent will be confirmed with the patient (and documented in the patient’s medical record) before these samples are taken.

The process for PK sampling for this trial will be included in the laboratory manual if the decision is made to include this research sampling.

### 5.1.12 Concomitant medications

Routinely used medications for the alleviation/treatment of symptoms of COVID-19 are permitted. Only approved anti-viral therapies to treat SARS-CoV-2 will be permitted. 

Any other medication (including over the counter or prescription medicines, vitamins, and/or herbal supplements) that the patient is receiving at the time of enrolment (including screening) or receives during the study must be recorded in the eCRF along with: 

- Reason for use. 

- Dates of administration including start and end dates. 

- Dosage information including dose and frequency. 

### 5.1.13 Trial partners

Patients will be asked to nominate a Trial Partner that the clinical research team can contact if the patient cannot respond during the trial period e.g. if the patient is sick at home, or because they are in hospital or have died. This is not a requirement of trial participation but a proposed way of supporting the patient’s participation on the trial.

### 5.1.14 Expenses and benefits

Reasonable travel expenses for any visits additional to normal care will be reimbursed, although no such additional visits are envisaged to be required.

# 6 patient selection

During the pilot phase, there will be ongoing review of the emerging data to inform adaptive decision making during the pilot and in support of further adaptations required for the continuation phase. Patient enrichment strategies may be implemented e.g. revision to inclusion criteria regarding co-morbidities, neutrophil/lymphocyte ratios, age.

## Inclusion Criteria

1. Patient willing and able to give informed consent.

2. Adults, 18 years of age and above who score moderate to very high risk according to COVID-age risk calculation (Appendix 4)

3. Typical symptoms of COVID-19 infection e.g. as per Public Health England guidance or equivalent organisations in the UK, Health Protection Scotland, Public Health Wales, Public Health Agency (Northern Ireland).

4. Evidence of current COVID-19 infection from a validated assay.

## Exclusion Criteria

The patient may not enter the trial if ANY of the following apply:

1. Significant electrolyte disturbance (e.g. hyperkalaemia, potassium >5.0 mmol/L).

2. Alanine aminotransferase (ALT) and/or aspartate aminotransferase (AST) and/or alkaline phosphatase (ALP) ≥ 2.5 x ULN.

3. Any condition that, in the Investigator's opinion, will prevent adequate compliance with trial therapy e.g. mild cognitive impairment (unable to follow instructions for self-assessment readings as assessed by the Investigator).

4. Patients on long term supplementary oxygen requirement (patients for whom hospital admission would not be considered e.g. care plan in the community is in place, are not excluded). 

5. Known hypersensitivity to camostat. 

6. Platelet count <100 x 10^9^/L.

7. Co-enrolment with a Clinical Trial of an Investigational Medicinal Product (CTIMP) will not be permitted. Co-enrolment with a clinical investigation of a Medical Device or a non-interventional clinical study will be considered on a study-by-study basis and in discussion with the relevant Chief Investigators and Sponsors and industrial collaborators. 

8. Co-enrolment involving non-interventional research (including questionnaire or tissue only studies) will be allowed provided this is not expected to affect the outcomes of both studies or place undue burden upon participants and their families. 

9. Female patients who are able to become pregnant (or are already pregnant or lactating). However, those patients who are of child bearing potential and have a negative serum or urine pregnancy test before enrolment and agree to use two forms of contraception (one effective form plus a barrier method [oral, injected or implanted hormonal contraception and condom; intra-uterine device and condom; diaphragm with spermicidal gel and condom]) or agree to sexual abstinence*, effective from the first administration of camostat, throughout the trial and for 28 days afterwards are considered eligible.

(*Abstinence is only considered to be an acceptable method of contraception when this is in line with the preferred and usual lifestyle of the subject. Periodic abstinence (e.g. calendar, ovulation, symptothermal, post-ovulation methods) and withdrawal are not acceptable methods of contraception.)

10. Male patients with partners of child-bearing potential (unless they agree to take measures not to father children by using a barrier method of contraception [condom plus spermicide] or to sexual abstinence* effective from the first administration of camostat, throughout the trial and for 28 days afterwards. Men with partners of child-bearing potential must also be willing to ensure that their partner uses an effective method of contraception for the same duration for example, hormonal contraception, intrauterine device, diaphragm with spermicidal gel or sexual abstinence). Men with pregnant or lactating partners must be advised to use barrier method contraception (for example, condom plus spermicidal gel) to prevent exposure of the foetus or neonate.

(*Abstinence is only considered to be an acceptable method of contraception when this is in line with the preferred and usual lifestyle of the subject. Periodic abstinence (e.g., calendar, ovulation, symptothermal, post-ovulation methods) and withdrawal are not acceptable methods of contraception.).  

11. Significant cardiovascular disease (as assessed via the participant’s medical record and history) as defined by: 

a. History of congestive heart failure requiring therapy (New York Heart Association [NYHA] III or IV – Appendix 3). 

b. History of unstable angina pectoris or myocardial infarction up to 6 months prior to trial entry. 

c. Presence of severe valvular heart disease. 

d. Presence of a ventricular arrhythmia requiring treatment. 

10. Known allergic reactions to components of camostat e.g., lactose intolerance (see Section 7.2.1).

# 7 pharmaceutical INFORMATION

Please see IB supplement and SmPC for further information if required.

## Supply of Investigational Medicinal Product

A complete Certificate of Analysis (CoA) and a Qualified Person (QP) certification must be provided with each delivery of camostat.

For information on camostat and re-ordering of supplies, contact the Clinical Research Associate (CRA)/Clinical Study Manager (CSM) responsible for the trial who will arrange further supplies.

Camostat will be supplied by: 

Fisher Clinical Services U.K. Limited

Langhurstwood Road

Horsham

RH12 4QD

The Manufacturing Organisation (Fisher Clinical Services U.K. Limited) must provide confirmation of the shipment to the CSM/CRA on despatch of the investigational medicinal product (IMP).

The primary and secondary packaging for the IMP will be labelled according to Eudralex Volume 4: Annex 13 ‘Manufacture of Investigational Medicinal Products’ of the European Union guide to Good Manufacturing Practice (GMP).

Upon arrival at site, patient ID must be added to the applicable variable fields on the primary and secondary labels. 

An example of the approved label can be found in the Trial Master File (TMF).

## Pharmaceutical Data

### 7.1.1 Formulation of camostat

### Camostat (camostat mesilate marketed as [FOIPAN®]) will be supplied as 100mg tablets.

Other ingredients as follows: Hydroxypropylcellulose, Carmellose calcium, Magnesium Stearate, Polyoxyethylene (105), polyoxypropylene (5) glycol, Lactose hydrate.

### 7.1.2 Storage conditions

All supplies must be stored in a secure, limited access storage area in its original packaging. Camostat must be stored at room temperature.

### 7.1.3 Stability and labelling of camostat

The tablets are supplied in boxes containing 5 blister packs. Patients should be instructed to store camostat at room temperature and out of sight and reach of children.

Labelling applied by pharmacy for camostat must include the following information:

Name of IMP; dose; expiry time/date; patient ID (and any other local requirements e.g. Pharmacy batch number).

NB: Any Pharmacy applied labels must not obscure the Annex 13 labels applied by the Manufacturing Organisation. Example labels should be filed in the TMF and approved by the Sponsor.

### 7.1.4 Dispensing of camostat

Sufficient tablets of camostat must be dispensed on each occasion to cover the prescribed dose for the full treatment period i.e. Days 1-14.

Patients should be supplied with the required quantity of camostat in the original boxes to cover the prescribed dose. Blister packs must not be cut to reduce tablet numbers at dispensing.

The tablets are supplied in blister packs and must not be removed from the primary packaging (provided to the patient) and placed in any other container. Tablets should be stored at room temperature and out of sight and reach of children.

### 7.1.5 Camostat administration

For patients randomised to the treatment arms:

Before dispensing the camostat tablets, the exact dosage must always be double-checked by a second suitably qualified person. All checks and double-checks must be documented (signed and dated) and the documentation must be available for the CRA/CSM to verify.

The camostat tablets must be swallowed whole (with water) and not chewed, crushed, dissolved or divided. Patients will be provided with instructions for when they should take their camostat.

Patients will be asked to take their camostat four times a day, one hour before each meal. After the patient has finished eating, they should wait approximately three hours before taking the next dose. Again, the patient should wait one hour after dosing before they can eat. The patient should aim to take their last dose of camostat before they go to bed.

Patients should be advised that there should be approximately a four-hour interval between doses, except between their last dose of the day and the next dose the following morning; this overnight interval will be longer. 

Should a patient miss a scheduled dose in error, for example, forgetting to take the dose, then the patient can take that delayed dose within two hours of the scheduled dose (but will need to refrain from eating for an hour afterwards). Subsequent doses will be delayed accordingly. 

The patient will be supplied with a diary card and asked to record each dose (time and dose) and timing of each meal. 

For dose modification guidance, see Section 5.4.2.

### 7.1.6 Camostat accountability

Accurate records of all IMP shipments, tablets dispensed, and all IMP returned must be maintained. This inventory record must be available for inspection at any time by CRAs or CSMs of the Sponsor. Investigational Medicinal Product supplies are to be used only in accordance with this protocol and under the supervision of the Investigator.

The Investigator undertakes not to destroy any unused or returned IMP unless authorised to do so by the Sponsor. Any unused IMP must be destroyed according to hospital procedures and properly accounted for using the IMP Destruction Form and on the IMP Accountability Record. During the course of the trial the CRA will check the numbers of camostat tablets shipped to the centre, the number used, and the number destroyed or returned. The pharmacy will give an account of any discrepancy.

### 7.1.7 Camostat supply to primary and secondary care pharmacies

For trial sites affiliated to a hospital Trust/Board, camostat will be supplied to the hospital pharmacy and maintained by the clinical trial pharmacists. 

If trial sites are opened where camostat is to be held in secondary pharmacies e.g. GP pharmacies, the Sponsor will perform a risk assessment to confirm the process by which camostat is managed and dispensed to patients and document this in the risk assessment and site management plan. 

# 8 Trial procedures

## Baseline assessments

It is expected that patients will have received a positive COVID-19 test result via oropharyngeal/nasopharyngeal swab (or other validated method) prior to further baseline assessments being performed and that this method will also be used for subsequent swab tests during the trial. Other validated methodology may be used during the course of the trial e.g. saliva test, to improve the patient experience and assuming equal to or improved test validation and sensitivity.

When the patient has indicated their interest in the trial as per Section 9, the following information will be ascertained by the clinical research team member via an initial video call (or phone) conversation: 

- Demographics – date of birth, gender.

- Height and weight.

- Symptoms of COVID-19, ancillary symptoms and length of symptoms – e.g. fever (≥ 37.8°C), persistent dry cough, muscle pain, shortness of breath, diarrhoea, headache, anosmia, other symptoms.

- Other potential risk factors including smoking (pack year history), ethnicity, occupation, symptomatic household (or other) contact, other relevant medication history.

- Previous medical history – particularly chronic disease (specifically hypertension, cardiovascular disease, diabetes mellitus, hyperlipidaemia, cancer and existing respiratory conditions), history of influenza vaccination (past two years).

If the above assessments are supportive of a home visit to conclude the below eligibility assessments, the patient will be asked to provide consent as per Section 9.2. The following will then be performed:

- Biochemical and haematological investigations to include:

- Haematology – haemoglobin (Hb), white blood cells (WBC), neutrophils, lymphocytes, and platelets.

- Biochemistry – sodium, potassium, magnesium, adjusted calcium, phosphate, urea, creatinine, total protein, albumin, bilirubin, alkaline phosphatase (ALP), alanine aminotransferase (ALT), aspartate aminotransferase (AST), clotting- prothrombin time (PT), activated partial thromboplastin time (APTT), gamma-glutamyl transferase (GGT), troponin, D-dimer.

- Not all of these parameters will be measured at the time and patients will be asked to consent to storing of blood samples for future research and analysis e.g. C-reactive protein (CRP), ferritin, B-type Natriuretic Peptide (BNP).

- Blood sample for antibody test (patients will be asked to consent to storing of blood samples for future research).

- Pregnancy test for women of childbearing potential.

- Fingertip peripheral oxygen saturation at rest (rest defined as sitting for at least 10 mins) and pulse rate.

- Body temperature (digital) (accepting antipyretic use as a potential confounder).

- Documentation of supplementary oxygen administration/ high-flow oxygen device. 

- Functional status (as per the Chelsea Critical Care Assessment Tool: Appendix 1.0).

- COVID-19 symptom collection using the Flu-iiQ questionnaire (see Appendix 2.0).

- Ordinal scale status assessment (as per secondary endpoint).

- During this process the potential patient will be asked, if possible, to include a phone number and email address for a Trial Partner, who may be contacted by the clinical research team if the patient is unavailable

Radiological investigations over normal clinical care are not required as part of this trial, but if performed as part of clinical care, chest x-rays and chest CT scans will be assessed and included in analysis by accessing the secondary care patient records. 

Additional laboratory investigations or procedures performed as part of clinical care during the trial period will be included in the analysis.  

## Confirmation of eligibility

Patients with a positive COVID-19 result and who are eligible according to the inclusion/exclusion criteria (post review of the assessments performed during baseline assessments), will be contacted by the clinical research team.  They will be randomised by the clinical research team and informed of their allocated group.

## On study assessments/procedures

**All patients** will be contacted as follows:

### 8.1.1 Daily for video call (or phone) monitoring: Days 1-14

- Temperature (digital) (accepting antipyretic use as a potential confounder).

- Fingertip peripheral oxygen saturation levels at rest (rest defined as sitting for at least 10 mins) and pulse rate.

Patients will be asked to take their temperature, pulse rate and oxygen saturation levels, twice daily so approximately 12 midday and 6pm.

- Saliva test if available (for batch analysis). 

Number of patients asked to perform this test and days of testing may be adapted based on test availability.

- COVID-19 symptom collection using the Flu-iiQ questionnaire (see Appendix 2.0).

- Ordinal scale status assessment (as per secondary endpoint).

For the pilot phase: The video (phone) calls made to the patient may be recorded for quality assurance purposes. Patients will be asked to consent to recording of their calls during the main consenting process and at the beginning of each call, the patient will be asked to verbally confirm their consent. These recordings will be emailed immediately afterwards to a secure NHS email address where they will be stored securely on the NHS server until the end of the pilot phase. See Section 14.3.1. This is not a requirement of trial participation but a proposed way of supporting the patient’s participation on the trial.

### 8.1.2 Home visit consultation with clinical professional: Days 7 and 14

The intention is that home visits occur on Days 7 and 14 to take research bloods and repeat swab.  However, this requirement may change if patients are able to provide samples by visiting the hospital (or other suitable clinical site) but only in accordance with government and clinical advice. If the Sponsor and site decide that sampling is not required, this will be communicated to the patient.  The Days 7 and 14 video (phone) calls will still go ahead as planned regardless of sampling.

- Oropharyngeal/nasopharyngeal swabs will be taken for repeat PCR for COVID-19 testing (or other COVID-19 validated assay).

- Biochemical and haematological investigations to include:

- Haematology – Hb, WBC, neutrophils, lymphocytes, and platelets.

- Biochemistry – sodium, potassium, magnesium, adjusted calcium, phosphate, urea, creatinine, total protein, albumin, bilirubin, ALP, ALT, AST, clotting-PT, APTT, GGT, troponin, D-dimer.

- Not all of these parameters will be measured at the time and patients will be asked to consent to storing of blood samples for future research and analysis e.g. CRP, ferritin, BNP.

- Raised parameters will be managed as per usual Trust clinical management. If results indicate that the patient should be admitted, the clinical research team will contact the patient and arrange for them to come into hospital for further assessment. For dose interruption/withdrawal criteria, see Section 5.4.2.1.Functional status where possible (as per the Chelsea Critical Care Assessment Tool: Appendix 1.0).

- Blood sample for antibody test (patients will be asked to consent to storing of blood samples for future research).

### 8.1.3 Video call (or phone) monitoring: Days 21 and 28

- COVID-19 symptom collection using the Flu-iiQ questionnaire (see Appendix 2.0).

- Ordinal scale status assessment – As per secondary endpoint.

- Temperature (digital) (accepting antipyretic use as a potential confounder).

- Peripheral oxygen saturation levels at rest (rest defined as sitting for at least 10 mins) and pulse rate.

For the pilot phase: The video (phone) calls made to the patient may be recorded for quality assurance purposes. Patients will be asked to consent to recording of their calls during the main consenting process and at the beginning of each call, the patient will be asked to verbally confirm their consent. These recordings will be emailed immediately afterwards to a secure NHS email address where they will be stored securely on the NHS server until the end of the pilot phase. See Section 14.3.1. This is not a requirement of trial participation but a proposed way of supporting the patient’s participation on the trial.

### 8.1.4 Days 1-28

Review of hospital and primary care records to evaluate use of any form of supplementary oxygen, CXR/ CT reports or images to assess progression or evolution of pneumonia and evidence of pulmonary thromboembolic events plus documentation of death (COVID-19 related mortality) during trial period.

Patients who are hospitalised will be followed up following discharge. At Day 28 post discharge, the patient will be contacted to collect information on existing/new symptoms and any additional results from COVID-19 testing.

### 8.1.5 Long term follow up

If the patient consents, they will be followed up for time to recovery or survival up to 1-year post enrolment.  This information will be obtained via hospital and primary care records, via the GP or by contacting the patient.

## Sample collection and transport

Local arrangements for collection of samples taken at home may vary depending on the trial centre and feasibility but may include home visits by the clinical research team, or patients attending Primary Care COVID hub clinics or drive-through testing centres. This will be captured in the trial and site risk assessment.

## Escalation of patient management

Patients will be informed of peripheral oxygen saturation thresholds, indications of progressive breathlessness, or other concerning symptoms (safety rule set developed by the TMG) that should trigger immediate self-referral to seek health advice from the appropriate channels as they normally would, according to urgency e.g. GP or dialling NHS 111 or 999. 

- As well as the safety rules being provided to the patient, these rules will also be provided to the clinical research team making the call to the patient. 

- Patients will be able to self-refer as per above however in the absence of patient action in response to a threshold being met, when the clinical researcher becomes aware during the call, this will trigger the clinical researcher to remind the patient of the safety rule set and their self-referral as needed. 

- Data collected from each call will be entered on the same day by the clinical researcher and therefore be available to the PIs for review on a daily basis. 

Daily video call (or phone) assessments will support reinforcement and safety netting of these safety rules, allowing for self or clinical research team referral for appropriate primary care, 111 or 999 review, in accordance with local clinical pathways. These calls to the patient will follow a set script to ensure consistent data collection and will take place at a consistent time for patient convenience but also at a time which will allow sufficient time for the Principal Investigator and/or Co-Investigator to review the data and to therefore contact the patient as needed. 

Any escalation of patient management, triggered by safety signals experienced by a patient, the clinical research team will also notify the Sponsor.

Patients requiring hospital admission will have clinical investigations performed in line with clinical care. Patients requiring hospital admission will continue to take camostat unless contraindicated by clinical condition or other clinical care. Patients will be provided with a trial ID card.

For dose interruption/withdrawal criteria, see Section 5.4.2.1.

**Table 1: Schedule of Assessments**

|  |  |  |  |  |  |  |  |  |  |  |
| --- | --- | --- | --- | --- | --- | --- | --- | --- | --- | --- |
|  |  | **Screening (& randomisation if eligible)** | **Day 1** | **Days 1-7** | **Day 7** **(+/- 1 day)** | **Days 8-14** | **Day 14** **(- 2 days)** | **Day 21** | **Day 28** | **Long term follow up: up to 1-year post enrolment** ^**E**^ |
| **Assessment** | Testing site | Home or at site (hospital or COVID hub) | Home visit | Daily video call (or phone) | Home visit | Daily call | Home visit | Video call (or phone) | Video call (or phone) | Medical records |
| **Oropharyngeal swab** **(or other validated method)** | X | Confirmed +ve result |  |  | x^**a**^ |  | X^**a**^ |  |  |  |
| **Blood (FBC& U&Es & other markers)** |  | X |  |  | x^**a**^ |  | X^**a**^ |  |  |  |
| **Blood – research and antibody test** |  | X |  |  | x^**a**^ |  | X^**a**^ |  |  |  |
| **Pregnancy test for women of childbearing potential** |  | X |  |  |  |  |  |  |  |  |
| **COVID-19 saliva test (if available)** ^**B**^ |  | X | X | x | x^**a**^ | X | X^**a**^ |  |  |  |
| **Demographics** **Height, weight** **Medical history** **Risk factors** **COVID-19 symptoms check** |  | X |  |  |  |  |  |  |  |  |
| **Delivery of trial pack (thermometer, oximeter)** ^**F**^ |  |  | X |  |  |  |  |  |  |  |
| **Delivery of camostat** ^**C**^ |  |  | X |  |  |  |  |  |  |  |
| **Camostat administration** ^**F**^ |  |  | X (first dose) | X | X (if patient recovered, discontinue camostat) | X | X  (last dose) |  |  |  |
| **Temperature (digital)** ^**D**^ |  | x | x | X | X | X | X | X | X |  |
| **Fingertip peripheral oxygen saturation and pulse rate** ^**D**^ |  | x | x | X | X | X | X | X | X |  |
| **Documentation of supplementary oxygen administration/ high-flow oxygen device** |  | x | x | x | x | X | X |  |  |  |
| **Assessment of:** **COVID-19 symptom collection (Flu-iiQ)** **functional status** ^**G**^ **(Chelsea score) and** **ordinal scale status assessment** |  | x | x | X | x | X | x | X | X |  |
| **Call with clinical researcher** |  |  |  | x | X | x | X | x | x |  |
| **Review of hospital records or primary care records** |  |  |  |  |  |  |  |  |  | X (performed by clinical research team) |

A. The intention is that home visits, by a clinical professional, occur on Days 7 and 14 to take research bloods and repeat swab.  However, this requirement may change if patients are able to provide samples by visiting the hospital (or other suitable clinical site). If the Sponsor and site decide that sampling is not required, this will be communicated to the patient.  The Day 7 and 14 video (phone) calls will still go ahead as planned regardless of sampling.

B. Saliva (viral load) test (if available), for batch analysis. Number of patients asked to perform this test and days of testing may be adapted based on test availability. 

C. If randomised to the camostat arm, patients will also receive camostat tablets to last for 14 days.

D. Two readings a day, ~12pm and then again at ~6pm.

E. In addition to the 1 year follow up, patients who are hospitalised will be followed up at Day 28, post discharge (to collect information on existing/new symptoms and also any additional results from COVID-19 testing).

F. Trial packs may be delivered by courier and for those patients randomised to the treatment arm, camostat may also be delivered by courier.

G. Functional status (where possible) will only be collected at Screening, Days 7 and 14.

# 9 Recruitment

## Identification of patients

The protocol is deliberately flexible so that it is suitable for several settings from which patients could be identified and recruited.  These settings can include but are not limited to:

- COVID hubs (GP practices or walk-in centres)

- Drive through testing sites

At the time of initial regulatory submission, the approach for community/outpatient testing was, for those patients with mild symptoms, to stay at home and self-isolate, and only present to hospital/consult NHS 111 if symptoms got worse. Testing availability was also changing on a regular basis in the UK.  However, testing is expected to expand rapidly and guidance for those with mild symptoms is also subject to change, especially if patients are in a high-risk group for more severe disease.

Sites with confirmed patient pathways for testing (and collection of results) and able to identify patients for out-patient support, will be selected in the first instance to support trial recruitment. In parallel, the Sponsor continues to liaise with primary care and hospital clinicians for regional variations in how patients are being tested and therefore will open further sites once there is clarity regarding the recruitment process. These sites will be submitted as a substantial amendment for appropriate regulatory approval. The Sponsor will document the patient recruitment process for each site in site management plans 

Risk assessments will be performed for each site selected to ensure appropriate monitoring, training and consenting processes are in place to ensure protection of the patient’s rights, safety and well-being.

As testing availability evolves across the UK, further sites will be confirmed in the primary care and hospital settings. These site management plans will be held in the TMF.

### 9.1.1 Testing at COVID hubs (GP practices or walk-in centres)

Patients who suspect they have COVID-19 should dial 111 and thereafter, will be referred to a GP/COVID hub/hospital for testing. Patient information material will be available to the patient in the form of a leaflet which can direct them to a trial specific website for further information. The leaflet will contain contact details of the associated (regional) clinical trial team affiliated with a hospital Trust/Board. 

If the patient results confirm COVID-19 infection, the patient can contact the clinical trial team to register their interest in participating in the trial. At this point, the main patient Information Consent Document (ICD) can be emailed to the patient and the trial can be discussed with the patient with opportunity to ask questions via a video (phone) call. The patient may also undertake basic screening questions with the clinical research team.

If the patient wishes to consent to the trial, the patient will be asked to give verbal consent over the video (phone) call, with the researcher signing the consent form at that point with the patient on the call. To complete screening assessments, blood samples and assessments are required which require a face to face visit. The intention is that community research nurses will attend to the patient in the home. Consent will be reaffirmed before any trial procedures take place and a paper version of the ICD will be signed at the earliest opportunity, by the patient which they will then keep (to avoid risk from paper copies handled by people with infection). See Section 9.2.

If eligibility is confirmed, the patient will be randomised by the clinical trial team and the patient informed of whether they are on the treatment or control arm. All randomised patients will receive a trial pack (to include diary card, questionnaires, thermometers and oximeters) and for those patients on the treatment arm, camostat tablets to cover 14 days of treatment.  These packs will be couriered to the patient with direction for courier to safely deliver the package whilst maintain safe social distancing. The clinical trial team will call the patient to ensure the patient is clear with the instructions on how to use the contents of the pack.

If the COVID hubs change practice or if demand for on-site recruitment is challenging, the above workflow may be changed after review by the TMG.

### 9.1.2 Other settings

Other agencies/organisations who receive calls from the public e.g. NHS 111, COVID hot hubs and Accident and Emergency departments, may be able to provide leaflets, advertise posters, which direct potential patients to the clinical trial team for more information.

## Informed Consent

Written and/or verbal versions of the ICD will be presented to the patients detailing the exact nature of the study; what it will involve for the patient; the implications and constraints of the protocol; and any risks involved in taking part. It will be clearly stated that the patient is free to withdraw from the study at any time for any reason without prejudice to future care, without affecting their legal rights, and with no obligation to give the reason for withdrawal.

Given the acute nature of COVID-19 and the potential for rapid progression of symptoms, patients will be asked to consider their decision over 12 hours as camostat (if randomised to that arm) should be given within 24 hours of presentation if possible.  However, patients will be given further time if required. 

Wherever possible the patient will be provided with a copy of the ICD in a paper format.  If paper is not permitted, an electronic version of the ICD will be provided that the clinical research team will go through with the patient either in person or over the telephone or video link.

In whatever way the information is presented, the patient must be given adequate time to think about their commitment to the trial and to allow them to ask any questions about the trial. 

The patient may provide verbal consent under the current restrictions and be asked to sign a copy of the ICD (at the earliest opportunity) to be made available at a later date to the research team (this could take the form of a photograph which is sent to/taken by the clinical trial team).  This consent in whichever form must be documented fully by the person taking consent and the person taking consent should sign a consent form also.  Reconciling the two consent forms will take place later once restrictions are lifted, or if this is not possible, the documentation in the patient’s records will serve to show that consent had been obtained and the way in which consent was obtained. The notation in the patient’s record should also include a statement of why the ICD signed by the patient was not retained e.g. due to potential contamination of the document by infectious material).

It is acknowledged that usual research practice is for the researcher and the patient to both sign and date the same ICD, in person when together. However, given the nature of the COVID-19 outbreak and to minimise avoidable risks, the preceding process has been agreed and providing careful and thorough documentation, consent can be taken in this way. The patient’s GP will be made aware of their participation in the trial via letter.

# 10 Definition of End of Trial

It is the responsibility of the Sponsor to inform the MHRA and the REC within 90 days of the End of Trial (EoT) that the trial has closed.

In cases of early termination of the trial (for example, due to toxicity) or a temporary halt by the CDD, the CDD will notify the MHRA and the REC within 15 days of the decision and a detailed, written explanation for the termination/halt will be given.

Recruitment will cease when:

- Camostat is considered too toxic to continue before the required number of patients have been recruited.  

- The stated number of patients to be recruited has been reached.

- The stated objectives of the trial are achieved.

Regardless of the reason for termination, all data available for patients at the time of discontinuation of follow-up must be recorded in the electronic/case report form (e/CRF). All reasons for discontinuation of treatment must be documented.  

In terminating the trial, Investigators must ensure that provision is made to ensure appropriate ongoing care for the patient.

The End of Recruitment is defined as the date the last patient is recruited.

The End of Trial is defined as the date when the one-year survival data has been obtained for the last patient or the final follow-up visit (whichever is the latter). 

For analysis and reporting plans, please see Section 13.

To note: If camostat related SAEs are still ongoing at the EoT point, these will continue to be followed up as per protocol.

# 11 Discontinuation/ Withdrawal of PaTIENTs from Study Treatment

Each patient has the right to withdraw from the trial at any time.  In addition, the investigator may discontinue a patient from the trial at any time if the investigator considers it necessary for any reason including: 

- Ineligibility (identified retrospectively).

- Significant protocol deviation.

- Significant non-compliance with treatment regimen or trial requirements.

- Consent withdrawn*.

The reason for withdrawal will be recorded in the patient’s records and e/CRF. In the case of a patient in the control arm being withdrawn from the trial, attempts will be made to recruit a replacement. 

*A decision by a patient that they no longer wish to continue receiving trial treatment should not be considered to be a withdrawal of consent for follow-up. However, patients are free to withdraw consent for some or all aspects of the trial at any time if they wish to do so. In accordance with regulatory guidance, de-identified data that have already been collected and incorporated in the trial database will continue to be used (and any identifiable data will be destroyed).

# 12 SAFETY REPORTING

## Definitions

### 12.1.1 Adverse Event (AE)

An AE or adverse experience is:

An adverse event (AE) is any untoward, undesired or unplanned medical occurrence in a patient administered an investigational medicinal product (IMP), a comparator product or an approved drug. 

An AE can therefore be any unfavourable and unintended sign (including an abnormal laboratory finding), symptom or disease temporally associated with the use of the trial medication, whether or not considered related to the trial medication.

### 12.1.2 Adverse Drug Reaction (ADR)

All untoward and unintended responses to a medicinal product related to any dose.

The phrase "responses to a medicinal products" means that a causal relationship between a trial medication and an AE is at least a reasonable possibility, i.e., the relationship cannot be ruled out.

All cases judged by either the reporting medically qualified professional or the sponsor as having a reasonable suspected causal relationship to the trial medication qualify as adverse reactions.  

### 12.1.3 Serious Adverse Event (SAE)

A serious adverse event is any untoward medical occurrence that at any dose:

- Results in death

- Is life-threatening, NOTE: A "life-threatening" event is defined as an event when the patient was at substantial risk of dying at the time of the AE occurring; it does not refer to an event which hypothetically might have caused death if it were more severe.

- Requires in-patient hospitalisation where they are admitted for more than 24 hours or prolongs existing in-patient hospitalisation (although some hospitalisations are exempt from SAE reporting, i.e. hospital admissions planned prior to the patient entering the trial; overnight stays for planned procedures).

- Results in persistent or significant disability/incapacity, or

- Is a congenital anomaly/birth defect.

- Other important medical events. NOTE: A medically important event is defined as any event that may jeopardise the patient or may require intervention to prevent one of the outcomes listed above. Examples include allergic bronchospasm (a serious problem with breathing) requiring treatment in an emergency room, serious blood dyscrasias (blood disorders) or seizures/convulsions that do not result in hospitalisation. Development of drug dependency or drug abuse including overdose would also be examples of important medical events. Other events that may not result in death, are not life threatening, or do not require hospitalisation, may be considered a serious adverse event when, based upon appropriate medical judgement, the event may jeopardise the patient and may require medical or surgical intervention to prevent one of the outcomes listed above.

To ensure no confusion or misunderstanding of the difference between the terms "serious" and "severe", which are not synonymous, the following note of clarification is provided:

The term "severe" is often used to describe the intensity (severity) of a specific event (as in mild, moderate, or severe myocardial infarction); the event itself, however, may be of relatively minor medical significance (such as severe headache).  This is not the same as "serious," which is based on patient/event outcome or action criteria usually associated with events that pose a threat to a patient's life or functioning as defined in the bullet points above.  Seriousness (not severity) serves as a guide for defining regulatory reporting obligations.

### 12.1.4 Suspected Unexpected Serious Adverse Reaction (SUSAR)

A SUSAR is a suspected, unexpected serious, adverse reaction that is deemed related to IMP, where the nature of the adverse event or severity of the event is not consistent with the applicable product information (e.g. reference safety information). All AEs and SAEs will be assessed by CDD for seriousness, causality and expectedness. The Pharmacovigilance Department will expedite all SUSARs to the relevant Competent Authority/Authorities and the relevant Ethics Committee(s) within the timelines specified in legislation (SI 2004/1031 as amended). 

### 12.1.5 Urgent Safety Measures (USM)

The Sponsor or Investigator may take appropriate USMs in order to protect the patient of a clinical trial against any immediate hazard to their health or safety. This includes procedures taken to protect patients from pandemics or infections that pose serious risk to human health. 

USMs may be taken without prior authorisation from the competent authority. However, The Medicines and Healthcare products Regulations Agency (MHRA) and the Research Ethics Committee (REC) must be notified within three days of such measures being taken.

Should the site initiate a USM, the Investigator must inform the Sponsor immediately either by:

- email: sae@cancer.org.uk; or

- telephone: 020 3469 6905;

The notification must include the minimum information: 

- the date of the USM; 

- who took the decision; 

- what action has been taken and 

- why the action was taken.

The Sponsor will then notify the MHRA and the REC within three days of USM initiation.

## Causality 

The relationship of each adverse event to the trial medication must be determined by a medically qualified individual according to the following definitions:

| Highly probable | Starts within a time related to the IMP administration and No obvious alternative medical explanation. |
| --- | --- |
| Probable | Starts within a time related to the IMP administration and Cannot be reasonably explained by known characteristics of the patient’s clinical state. |
| Possible | Starts within a time related to the IMP administration and A causal relationship between the IMP and the AE is at least a reasonable possibility. |
| Unlikely | The time association or the patient’s clinical state is such that the trial drug is not likely to have had an association with the observed effect. |
| Not related | The AE is definitely not associated with the IMP administered. |

*Note: Drug-related refers to events assessed as possible, probable or highly probable.*

The Investigator must endeavour to obtain sufficient information to determine the causality of the AE (i.e. IMP, other illness etc.) and must provide his/her opinion of the causal relationship between each AE and IMP. This may require instituting supplementary investigations of significant AEs based on their clinical judgement of the likely causative factors and/or include seeking a further opinion from a specialist in the field of the AE.

The following guidance should be taken into account when assessing the causality of an AE:

- Previous experience with the IMP and whether the AE is known to have occurred with the IMP.

- Alternative explanations for the AE such as concomitant medications, concurrent illness, non-medicinal therapies, diagnostic tests, procedures or other confounding effects.

- Timing of the events between administration of the IMP and the AE.

- IMP blood levels and evidence, if any, of overdose.

- De-challenge, that is, if the IMP was discontinued or the dosage reduced, what happened to the adverse reaction?

- Re-challenge, that is, what happened if the IMP was restarted after the AE had resolved?

## Procedures for Recording Adverse Events

All AEs will be recorded in the eCRF.  

The following information will be recorded: patient ID including the patient trial number, age and gender; verbatim term for the AE description, date of onset and end date as well as outcome, severity of the event, assessment of relatedness to trial medication, other suspect drug or device and action taken.  Follow-up information should be provided as necessary. AEs should be recorded on the CRF from the date of written/verbal informed consent (whichever is the earlier) being given until they have completed the 28 days from randomisation follow up period. 

AEs considered related to the trial medication or COVID-19, as judged by a medically qualified investigator or the sponsor will be followed until resolution or the event is considered stable.  All related AEs that result in a patient’s withdrawal from the trial or are present at the end of the trial, should be followed up until a satisfactory resolution occurs.

It will be left to the investigator’s clinical judgment whether or not an AE is of sufficient severity to require the patient’s removal from treatment.  Although the Sponsor will hold the final decision if there is a disagreement in the management of the patient’s treatment.  A patient may also voluntarily withdraw from treatment due to what he or she perceives as an intolerable AE.  If either of these occurs, the patient must undergo an end of study assessment and be given appropriate care under medical supervision until symptoms of the AEs cease or the condition becomes stable.

The severity of events will be assessed on the following scale:  1 = mild, 2 = moderate, 3 = severe and as judged by a medically qualified investigator.  

## Procedures for recording pregnancies

Any pregnancy occurring during the clinical trial (within 28 days after the last dose of camostat) and the outcome of the pregnancy (including the partner of a male patient), should be recorded using a pregnancy notification form and followed up until an outcome is known. If the outcome of the pregnancy results in an SAE such as congenital abnormality or birth defect, an SAE should be reported in accordance with Section 12.5.

The Investigator should document within the patient notes, the patient confirming consent for the Sponsor to collect pregnancy follow-up information. In the case that the partner of a patient becomes pregnant, a consent form should be provided to the patient’s partner in order to obtain consent for collecting privacy data, in accordance with data protection regulation.

If a pregnancy is confirmed (for a female trial patient) whilst on the trial, the patient must be withdrawn from trial treatment.

## Reporting Procedures for Serious Adverse Events

For eligible patients, SAE collection and monitoring (as per risk assessed monitoring plan) will commence at the time the patient gives their written/verbal consent (whichever is the earlier) to participate in the trial and will continue for 28 days from randomisation. 

In addition to the SAE reporting criteria in Section 12.1.3,

During the trial period (Days 1-28):

- All hospitalisations as per the definition stated in Section 12.1.3 (regardless of causality) will be followed up until resolution, discharge or death.

- Patients who are hospitalised will also be followed up, 28 days after they are discharged. The patient will be contacted to collect information on existing/new symptoms and also any additional results from COVID-19 testing.

Following the trial period, up to the 1 year follow up timepoint, only SAEs related to camostat or COVID-19 are reportable. These will be followed up until resolution, discharge or death. 

Should an Investigator become aware of any IMP-related or COVID-19 related SAEs after this period, these must also be reported to the CDD within the expedited timelines. 

All SAEs regardless of causality must be reported to the Pharmacovigilance Department within 24 hours of becoming aware of the event. SAEs should be documented on an SAE report form, using the completion guidelines provided. SAE report forms should be emailed to the Pharmacovigilance Department mailbox: [sae@cancer.org.uk](mailto:sae@cancer.org.uk). The following information must be provided as a minimum:

- Patient identifiers including patient trial number and initials, age or date of birth and gender,  

- SAE term, onset date and causality assessment

- A description of the SAEs

- Dose and duration of the treatment with the medicine 

- The batch number of the medicine 

- Any other medications being taken at the same time including non-prescriptive medicines, herbal remedies and contraceptives

- Any other health conditions the patient has or may have

Follow-up of SAEs by the Pharmacovigilance Department will continue until the events resolve or stabilise. The Pharmacovigilance Department will make requests for further information on SAEs to the Investigators at regular intervals. Requested follow-up information should be reported to the Pharmacovigilance Department in a timely manner and as soon as possible after receipt of the follow-up request or within 24 hours of becoming aware of new/updated information. 

## Specific Events of Interest

The following events will be closely monitored as trial specific events of interest:

- Shock or anaphylactoid symptoms such as decreased blood pressure, dyspnoea and pruritis 

- Rash, pruritis 

- Gastrointestinal events (nausea, abdominal discomfort, abdominal distension)

- Changes in liver enzymes (AST, ALT, GGT) or jaundice

- Thrombocytopenia 

- Hyperkalaemia 

## Annual Development Safety Update Reports (DSUR)

In addition to the expedited reporting above, CRUK will submit a DSUR in accordance with ICH E2F to the Competent Authority (MHRA in the UK) and the REC.

# 13 data analysis and statistical considerations

## Description of statistical methods

All statistical aspects of the study will be fully documented in a comprehensive Statistical Analysis Plan authored by the study statistician and agreed by the independent Trial Steering Committee and the independent Data Monitoring Committee. 

Descriptive statistics will be used to describe the demographics for each randomised group and overall. For categorical data, frequencies will be reported, and for continuous variables means and standard deviations, or for skewed data, medians and interquartile ranges will be reported. 

The primary outcome is hospitalisation requiring supplemental oxygen (within 28 days of randomisation) (Y/N) a logistic regression will be used. Site will be fitted as a fixed effect if the number of sites is 5 or less, otherwise as a random effect (and hence a mixed effects logistic regression), and will adjust for pre-specified baseline covariates known to be strongly associated with the primary outcome, along with an indicator variable for randomised group to estimate the treatment effect S. Missing data is thought to be low (around 5%) but nonetheless the robustness of the findings to any patterns of missing data will be investigated using appropriate sensitivity type analyses, including multiple imputation under an assumption of missing at random.  Subgroup analyses on the primary outcome will include by (a) age (70-90 vs <70), (b) lymphopaenia change (<20% vs. >=20%), and (v) neutrophil:lymphocyte ratio (<2.18 vs. >= 2.18). 

The secondary outcome of COVID-19 related hospitalisation will be analysed as per the primary outcome. The secondary outcomes of (i) supplemental oxygen free days and (ii) ventilator free days (each determined in the period up to 28 days from randomisation) will be analysed as count data using a negative binomial regression model. The secondary outcome of the WHO 9-point ordinal scale (0=complete recovery, …, 8=death) will be analysed in a number of ways, including (i) time to worst point on the scale; and (ii) time to deterioration of two points, both using Cox proportional hazards regression models, or a restricted mean survival time (RMST) approach if non-proportionality does not hold; and (iii) the overall 0-8 scale using an ordinal regression analysis (proportional odds model); and (iv) any other exploratory categorisations of the scale e.g. 0=complete recovery (Y/N) at day 14, or ‘responders’ (any improvement by day 14). The secondary outcomes of time to mortality (i) either within 28 days or (ii) within 1 year, both from randomisation, will also be analysed using a Cox PH approach (or RMST). The secondary outcomes of time to apyrexia (maintained for 48 hours) and time to improvement by two points in peripheral oxygen saturation (measured in daily self-assessment in the fingertip) will also be analysed by Cox PH or RMST. The Flu-iiQ 25-item PROM will be analysed with an appropriate linear model. The viral load parameters (change from baseline at 7 and 14 days in respiratory log_10_ viral load for oropharyngeal swab RT-PCR; and salivary RT-PCR at days 1-14; and upper respiratory viral shedding at days 1-14) will be analysed using mixed effects repeated measures linear models; and time to clearance of nasal SARS-CoV-2 (defined by 2 consecutive nasal swabs by qPCR) using Cox PH or RMST. The exploratory outcomes based on biological & haematological parameters measured in venous blood at days 7 and 14 and venous samples RT-PCR at days 7 and 14 will be analysed using mixed effect repeated measures linear models as well.      

All enrolled patients who receive at least one dose of camostat will be evaluable for safety.

Safety data will be collected from the date of written consent. Safety variables will be summarised by descriptive statistics. 

Adverse events will be reported as tables of frequency of AEs by body system and by worst severity grade observed. Tables should indicate related and unrelated events

Adverse events will be reported for each arm and presented as tables of frequency of AEs by body system and by worst severity grade observed. Tables should indicate related and unrelated events. 

## The Level of Statistical Significance

## Sample size determination

For 90% power at a 5% level of significance, on the primary outcome of hospitalisation requiring supplemental oxygen (yes or no), assuming that in the enriched cohort the control rate will be 40%, and the in the camostat group that will reduce by an absolute 16% (or a relative reduction of 40%) to 24%, the study will require 177/group or 354 in total.  Adjusting this sample size for 1 interim analysis at 50% with mature primary outcome data using a group sequential design (Hwang-Shih-DeCani spending function with O’Brien Fleming bounds) increases this sample size to 370. If we assume that 5% will be lost to follow up on the primary outcome, the trial is required to recruit 370/0.95 = 389 participants. Therefore, the trial aims to recruit 100 in the pilot phase and up to a maximum of 289 in the continuation phase. 

## Subgroup analysis

Subgroup analysis of patients aged 70-90 will be performed as this patient group has a higher mortality in published literature. 

Subgroup analysis of patients with a 20% reduction in lymphocyte count will be performed as this patient group has a higher mortality in the published literature. 

Subgroup analysis of patients with a neutrophil: lymphocyte ratio greater than 2.18 will be performed as this patient group has a higher mortality in the published literature. 

## Interim reporting 

There will be a single analysis of the main study findings either at (a) the end of the pilot phase if the decision is not to proceed to the continuation study, or (b) at the formal interim analysis at approximately 50% of maximum sample size randomised and with full data on the primary outcome if the decision is to stop the study early due to either overwhelming evidence of benefit or for futility, or (c) if the decision at that interim analysis is to continue, then at the end of the study with full recruitment up to a maximum sample size of 389. 

Full details of the approaches to assessing the feasibility of continuing into the full study at the pilot study, and all the statistical details of the formal interim analysis at 50% will be included in the Statistical Analysis Plans for the independent Trial Steering Committee and the independent Data Monitoring Committee, respectively.

##  Clinical Study Report

At appropriate intervals, interim data listings will be prepared to give the Investigators the possibility to review the data and check the completeness of information collected. All clinical data will be presented at the end of the trial on final data listings. The Sponsor will prepare a Clinical Study Report (CSR) based on the final data listings. The report will be submitted to the Investigator(s) for review and confirmation it accurately represents the data collected during the course of the trial. Summary results of the trial will be provided by the Sponsor to the Regulatory Authority (MHRA) and to the Research Ethics Committee.

# 14 DATA MANAGEMENT

The plan for the data management of the trial are outlined below. Further details of the data management aspects of the study will be fully documented in a comprehensive Data Management Plan (DMP) authored by the trial Clinical Data Manager (CDM).

## Source data

Source documents are where data are first recorded, and from which patients’ eCRF data are obtained. These include but are not limited to: primary or secondary care records (from which medical history and previous and concurrent medication may be summarised into the CRF), clinical and office charts, laboratory and pharmacy records, diaries, microfiches, radiographs, and correspondence. For some data, the data will be recorded directly into the eCRF and therefore acts as both source and CRF. All documents will be stored safely in confidential conditions. On all trial-specific documents, other than the signed consent, the patient will be referred to by the trial patient number/code, initials, not by name.

The DMP will clearly state which data is entered directly into the eCRF and which data is derived from other source documents for entering.

## Access to data

Direct access will be granted to authorised representatives from the Sponsor, host institutions and the regulatory authorities to permit trial-related monitoring, audits and inspections.

## Data recording and record keeping

The eCRF for this trial will be a RAVE clinical database. As per Section 14.1, source data may be separate documentation/diaries/records, however the eCRF itself may also act as a source for certain data items. As per the DMP, source documents will be clearly stated and the Sponsor may ask that certified copies are scanned/posted as appropriate for source data verification in a timely fashion, as it is anticipated that on-site monitoring visits may not be possible due to restrictions at sites. 

The Investigator and/or Sponsor must retain copies of the essential documents for a minimum of 25 years following the end of the trial.

During the clinical trial and after trial closure the Investigator must maintain adequate and accurate records to enable both the conduct of a clinical trial and the quality of the data produced to be evaluated and verified. These essential documents (as detailed in Chapter V of Volume 10 (Clinical Trials) of The Rules Governing Medicinal Products in the European Union based upon Section 8 of the ICH GCP Guidelines), including source documents such as worksheets, scans, hospital records, diary cards, trial related documents and copies of the e/CRFs, associated audit trail and serious adverse event (SAE) report forms, shall show whether the Investigator has complied with the principles and guidelines of Good Clinical Practice (GCP).

All essential documents required to be held by the Investigator must be stored in such a way that ensures that they are readily available, upon request, to the Regulatory Agency or Sponsor, for the minimum period required by national legislation or for longer if needed by Sponsor. Records must not be destroyed without prior written approval from Sponsor.

The medical files of trial subjects shall be retained in accordance with national legislation and in accordance with the maximum period of time permitted by the hospital, institution or private practice.

### 14.1.1 Recording of daily calls

During the pilot phase, patients will be asked for permission and consent to record their daily video (phone) calls with the clinical trial team. Patients will be asked to consent during main trial consent and to also confirm consent verbally before each video/phone call begins. After each call is completed, the recording will immediately be emailed to a secure NHS email for storage on a secure NHS server. The recording will be deleted thereafter from the original recording device. Each NHS Trust/Board will be responsible for the security and confidentiality of these recordings. These recordings will not be shared with the Sponsor to protect the patient’s confidentiality. The NHS Trust/Board will be solely responsible for the recording, transfer, storage and deletion of the recording. All recordings will be deleted from the NHS secure server at the end of the pilot phase.

If recordings are intended to be continued into the continuation phase, the above arrangements will be applicable. 

This is not a requirement of trial participation but a proposed way of supporting the patient’s participation on the trial.

# 15 QUALITY CONTROL AND QUALITY ASSURANCE PROCEDURES 

## Risk assessment

A risk assessment and monitoring guidelines will be prepared before the trial opens and will be reviewed periodically throughout the trial to reflect significant changes to the protocol or monitoring activities.

## Monitoring 

In order to ensure that quality data is collected and that sites are adhering to the protocol, ICH GCP and other guidelines and local regulations, data will be monitored at regular intervals by the Sponsor.

The level of monitoring will be informed by the trial specific risk assessment and captured in the trial monitoring plan.

The Investigator and clinical trial teams will be responsible for entering trial data into the eCRF and providing certified copies of source data where applicable to facilitate source data verification. It is the Investigator’s responsibility to ensure the accuracy of the data entered into any source document provided to the Sponsor, and to ensure that any patient data provided to the Sponsor is suitably anonymised.

Once source documents have been completed by site staff they will be scanned and emailed to the Sponsor. Any missing data or any data that requires querying with the site staff will be raised by the Sponsor.

Movement of people may be restricted, therefore, monitoring of patient medical records will be completed remotely where possible in accordance with the trial-specific monitoring plan.

Monitoring of the data will occur as the data is being entered into the database.

In order to verify that the trial is conducted in accordance with ICH GCP, regulatory requirements, and the trial protocol, and that the data is authentic, accurate and complete, source data will be verified.

Upon trial completion, a Closedown Visit will be conducted.

## Quality assurance

The Sponsor will assess each trial site to verify the qualifications of each Investigator and the site staff and to ensure that the site has all of the required equipment. A Study Initiation meeting will occur where among other things the Investigator will be informed of their responsibilities and procedures for ensuring adequate and correct trial documentation. During this meeting, training will be provided to the investigator and the local study team, in accordance with the study-specific monitoring plan.

The Investigator is required to prepare and maintain adequate and accurate case histories designed to record all observations and other data pertinent to the trial for each patient. Trial data for each enrolled patient will be entered into the Source Worksheets by study site personnel.

##  Protocol deviations and amendments

The protocol should be adhered to throughout the conduct of the trial, if a situation arises where the conduct of the trial may not be in line with the protocol, then site should contact the Sponsor to discuss this.

Amendments to the protocol may only be made with the approval of the Sponsor. A protocol amendment may be subject to review by the assigned REC, Health Research Authority (HRA) and the MHRA. Written documentation of the Ethics Committee and HRA (and if appropriate the MHRA) ‘favourable opinion’ (i.e. approval) must be received before the amendment can be implemented and incorporated into the protocol if necessary.

##  Serious Breach of GCP

A serious breach is a breach which is likely to effect to a significant degree: the safety or physical or mental integrity of the subjects of the trial, or the scientific value of the trial.

In order that the Sponsor can fulfil their obligations in terms of reporting serious breaches of GCP to the MHRA within seven calendar days of identification, site staff must inform the Sponsor of any unplanned deviations to the trial protocol (or GCP principles) as soon as possible after the deviation occurs to allow prompt evaluation by the Sponsor.

# 16 ETHICal considerations

Before starting the trial, the protocol and ICD must receive the favourable opinion of the REC.

It is the Chief/Principal Investigator’s responsibility to update patients (or their authorised representatives, if applicable) whenever new information (in nature or severity) becomes available that might affect the patient’s willingness to continue in the trial. The CI/PI must ensure this is documented in the patient’s medical notes and the patient is re-consented.

The Sponsor and CI/PI must ensure that the trial is carried out in accordance with the GCP principles and requirements of the UK Clinical Trials regulations (SI 2004/1031 and SI 2006/1928 as amended), the ICH GCP guidelines and the WMA Declaration of Helsinki - Ethical Principles for Medical Research Involving Human Subjects adopted by the 18th WMA General Assembly, Helsinki, Finland, June 1964 and all subsequent amendments including Oct 2013.

## Approvals

The protocol, ICD and any proposed advertising material will be submitted as appropriate to the REC, regulatory authorities (MHRA in the UK), and host institution(s) for written approval.  

The Sponsor will submit and, where necessary, obtain approval from the above parties for all substantial amendments to the original approved documents.   

## Patient confidentiality

The trial staff will ensure that the patients’ anonymity is maintained.  The patients will be identified only by initials and patient ID number on worksheets and any electronic database. Patient names and telephone numbers will be stored securely at each NHS Trust/Board by the clinical trial team and will only be accessible by the clinical trial team at site.  All documents will be stored securely and only accessible by trial staff and authorised personnel. 

The collection and processing of personal data from the patients enrolled in this clinical trial will be limited to those data that are necessary to investigate the efficacy, safety, quality and usefulness of the drug used in this trial. The data must be collected and processed with adequate precautions to ensure patient confidentiality and compliance with applicable data privacy protection according to the applicable data protection regulations. The data collected will comply with the EU General Data Protection Regulation (GDPR) 2016/679 on the protection of individuals with regard to the processing of personal data.

For long term follow up, patients will be asked to explicitly consent to this on the ICD.

## Indemnity

This trial is sponsored by CR UK and therefore injury to a patient caused by the compounds under trial will not carry with it the right to seek compensation from the pharmaceutical industry. CR UK will provide patients with compensation for adverse side effects, in accordance with the principles set out in the Association of the British Pharmaceutical Industry (ABPI) guidelines on compensation for medicine-induced injury.

# 17 References

.

Arabi, Y. M. (2020). COVID-19: A Novel Coronavirus and a Novel Challenge for Critical Care. *Intensive Care Medicine*, March. https://doi.org/10.1007/s00134-020-05955-1.

Corner, E. W. (2012). The Chelsea Critical Care Physical Assessment Tool (CPAx): validation of an innovative new tool to measure physical morbidity in the general adult critical care population; an observational proof-of-concept pilot study. *Physiotherapy Journal*, https://www.physiotherapyjournal.com/article/S0031-9406(12)00028-4/fulltext.

Forrest. (1989). Tolerance and Pharmacokinetics of Camostat Mesylate (FOY-305) in Normal Human Subjects after Single Rising i.v. Infusion Doses. *European Journal of Clinical Pharmacology*, PP14.68 no. 36 (Suppl.).

Hoffmann. (2020). SARS-CoV-2 Cell Entry Depends on ACE2 and TMPRSS2 and is Blocked by a Clinically Proven Protease Inhibitor. *Cell* , 181, 1-10.

Hoffmann. (APR 2020). Nafamostat mesylate blocks 1 activation of SARS-CoV-2: New treatment option for COVID-19. *Anitmicrob. Agents Chemother.*, doi:10.1128/AAC.00754-20.

Iwata-Yoshikawa. (2019). TMPRSS2 Contributes to Virus Spread and Immunopathology in the Airways of Murine Models after Coronavirus Infection. *Journal of Virology*, 93: 6.

Kawase. (2012). Simultaneous Treatment of Human Bronchial Epithelial Cells with Serine and Cysteine Protease Inhibitors Prevents Severe Acute Respiratory Syndrome Coronavirus Entry. *Journal of Virology*, 86: 12.

Liu, C. Q. (2020). Research and Development on Therapeutic Agents and Vaccines for COVID-19 and Related Human Coronavirus Diseases. *ACS Central Science*, 6 (3): 315–31.

Matsuyama. (2010). Efficient Activation of the Severe Acute Respiratory Syndrome Coronavirus Spike Protein by the Transmembrane Protease TMPRSS2. *Journal of Virology*, 12658–12664.

Midgley. (1994). Metabolic Fate of 14C-Camostat Mesylate in Man, Rat and Dog after Intravenous Administration. *Xenobiotica; the Fate of Foreign Compounds in Biological Systems* , 24 (1): 79–92.

Ono Pharmaceutical Co., Ltd. (2020). *FOY-305-02 Pharmacokinetic Analysis Results (Draft report dated 21 August 2020).* 

Osborne, R. H. (2011). Development and Validation of the Influenza Intensity and Impact Questionnaire (FluiiQTM). Value in Health: . *The Journal of the International Society for Pharmacoeconomics and Outcomes Research*, 14 (5): 687-99.

Rabi. (2020). SARS-CoV-2 and Coronavirus Disease 2019: What We Know So Far. *Pathogens*, 9, 231.

Ruan, S. (2019). Likelihood of Survival of Coronavirus Disease 2019. . *The Lancet. Infectious Diseases,* , March. https://doi.org/10.1016/S1473-3099(20)30257-7.

Sato. (1992). A Dose-Finding Study of FOY-305 in Postoperative Reflux Esophagitis – A Double-Blind Comparative Study. *J Clin Ther Med.* 

Shirato, K. M. (2013). Middle East Respiratory Syndrome Coronavirus Infection Mediated by the Transmembrane Serine Protease TMPRSS2. *Journal of Virology*, 87 (23): 12552–61. https://doi.org/10.1128/JVI.01890-13.

Shulla. (2011). A Transmembrane Serine Protease Is Linked to the Severe Acute Respiratory Syndrome Coronavirus Receptor and Activates Virus Entry. *Journal of Virology,* , 873-882.

Yamamoto. (2016). Identification of Nafamostat as a Potent Inhibitor of Middle East Respiratory Syndrome Coronavirus S Protein-Mediated Membrane Fusion Using the Split-Protein-Based Cell-Cell Fusion Assay. *Antimicrobial Agents and Chmotherapy*, 6532-6539.

Yan. (2020). Structural basis for the recognition of SARS-CoV-2 by full-length human ACE2. *Science 367*, 1444-1448.

Zhou, Y. (2015). Protease inhibitors targeting coronavirus and filovirus entry. *Antiviral Res.*, http://dx.doi.org/10.1016/j.antiviral.2015.01.011.

# 18 appendices

## Appendix 1: The Chelsea Critical Care Physical Assessment tool (Cpax) – Copyright of Chelsea and Westminster NHS Foundation Trust (01MAR2010) (Corner, 2012)

| **Aspect of Physicality** | **Level 0** | **Level 1** | **Level 2** | **Level 3** | **Level 4** | **Level 5** |
| --- | --- | --- | --- | --- | --- | --- |
| **Respiratory Function** | Complete ventilator dependence. Mandatory breaths only.  May be fully sedated/ paralysed. | Ventilator dependence. Mandatory breaths with some spontaneous effort. | Spontaneously breathing with continuous invasive or non-invasive ventilatory support. | Spontaneously breathing with intermittent invasive or non-invasive ventilatory support **Or** continuous high flow oxygen (>15litres). | Receiving standard oxygen therapy (<15 litres). | Self-ventilating with no oxygen therapy. |
| **Cough** | Absent cough may be fully sedated or paralysed. | Cough stimulated on deep suctioning only. | Weak ineffective voluntary cough, unable to clear independently e.g. requires deep suction. | Weak, partially effective voluntary cough, sometimes able to clear secretions e.g. requires yanker suctioning. | Effective cough, clearing secretions with airways clearance techniques. | Consistent effective voluntary cough, clearing secretions independently. |
| **Moving Within the Bed e.g. rolling.** | Unable, maybe fully sedated/ paralysed. | Initiates movement. Requires assistance ≥ 2 people (maximal). | Initiates movement. Requires assistance≥1 person (moderate). | Initiates movement. Requires assistance 1 person (minimal). | Independent in ≥3 seconds. | Independent in <3 seconds. |
| **Supine to Sitting on the Edge of the Bed.** | Unable/ Unstable. | Initiates movement. Requires assistance ≥ 2 people (maximal). | Initiates movement. Requires assistance≥1 person (moderate). | Initiates movement. Requires assistance 1 person (minimal). | Independent in ≥3 seconds. | Independent in <3 seconds. |
| **Dynamic Sitting (i.e. when sitting on the edge of the bed/unsupported sitting)** | Unable/ Unstable | Requires assistance ≥2 people (maximal). | Requires assistance≥1 person (moderate). | Requires assistance 1 person (minimal). | Independent with some dynamic sitting balance, i.e. able to alter trunk position within base of support. | Independent with full dynamic sitting balance, i.e. able to reach out of base of support. |
| **Standing Balance** | Unable/ unstable/ bedbound. | Tilt table or similar | Standing hoist or similar. | Dependant on frame, crutches or similar. | Independent without aides. | Independent without aids and full dynamic standing balance, i.e. able to reach out of base of support. |
| **Sit to Stand** (Starting position: ≤ 90 degrees hip flexion) | Unable/ Unstable. | Sit to stand with maximal assistance e.g. standing hoist or similar. | Sit to stand with moderate assistance e.g. 1-2 people. | Sit to stand with minimal assistance e.g. 1 person. | Sit to stand independently pushing through arms of the chair. | Sit to stand independently without upper limb involvement. |
| **Transferring from Bed to Chair.** | Unable/ Unstable. | Full hoist. | Standing hoist or similar. | Pivot transfer (no stepping) with mobility aid or physical assistance. | Stand and step transfer with mobility aid OR physical assistance. | Independent transfer without equipment. |
| **Stepping** | Unable/ Unstable. | Using a standing hoist, or similar. | Using mobility aids AND assistance > 1 person (moderate). | Using mobility aid AND assistance 1 person (minimal). | Using mobility aid OR assistance 1 (minimal). | Independent without aid. |
| **Grip Strength** (predicted mean for age and gender on the strongest hand.) | Unable to assess - patient self-assessment at home |  |  |  |  |  |

## Appendix 2: COVID-19-symptom collection using the Influenza Intensity and Impact Questionnaire (FluiiQ™) - provided free of charge by Measured Solutions for Health and tailored for COVID-19 trials. 

##  

**1. Since this time yesterday, because of COVID-19 have you had the following symptoms?**

|  | **None** | **Mild** | **Moderate** | **Severe** |
| --- | --- | --- | --- | --- |
| a. Cough | □ | □ | □ | □ |
| b. Sore throat | □ | □ | □ | □ |
| c. Headache | □ | □ | □ | □ |
| d. Nasal congestion | □ | □ | □ | □ |
| e. Fever… | □ | □ | □ | □ |
| f. Body aches… | □ | □ | □ | □ |
| g. Fatigue… | □ | □ | □ | □ |
| h. Neck pain | □ | □ | □ | □ |
| i. Interrupted sleep | □ | □ | □ | □ |
| j. Wheezing | □ | □ | □ | □ |
| k. Expectoration… | □ | □ | □ | □ |
| l. Shortness of breath | □ | □ | □ | □ |
| m. Loss of appetite | □ | □ | □ | □ |
| n. Loss of smell | □ | □ | □ | □ |
| o. Nausea | □ | □ | □ | □ |
| p. Loss of taste | □ | □ | □ | □ |

2. **Since this time yesterday, has COVID-19 infection affected your ability to:**

|  | **No Difficulty** | **Some Difficulty** | **Moderate Difficulty** | **Great Difficulty** |
| --- | --- | --- | --- | --- |
| a. Get out of bed | □ | □ | □ | □ |
| b. Prepare meals… | □ | □ | □ | □ |
| c. Perform usual… | □ | □ | □ | □ |
| d. Go out of the room… | □ | □ | □ | □ |
| e. Concentrate on… | □ | □ | □ | □ |
| f. Take care of… | □ | □ | □ | □ |

3. **Since this time yesterday, has the COVID-19 infection made you:**

|  | **Not at all** | **Somewhat** | **Moderately** | **Extremely** |
| --- | --- | --- | --- | --- |
| a. Irritable… | □ | □ | □ | □ |
| b. Feel helpless | □ | □ | □ | □ |
| c. Worried | □ | □ | □ | □ |
| d. Frustrated | □ | □ | □ | □ |

* some items have been truncated due to copyright.

Influenza Intensity and Impact Questionnaire (Flu-iiQTM) ©RH Osborne (2006, 2022). No part of the Flu-iiQTM may be copied or reproduced in any form without written permission from the author: [info@measuredsolutions.com.au](mailto:info@measuredsolutions.com.au)

## Appendix 3: New York Heart Association (NYHA) scale 

| Class I – | Patients with cardiac disease but without resulting limitation of physical activity; ordinary physical activity does not cause undue dyspnoea (or fatigue, palpitation or anginal pain). |
| --- | --- |
| Class II – | Patients with cardiac disease resulting in slight limitation of physical activity; they are comfortable at rest; ordinary physical activity results in dyspnoea (or fatigue, palpitation or anginal pain). |
| Class III – | Patients with cardiac disease resulting in marked limitations of physical activity; they are comfortable at rest; less than ordinary physical activity causes dyspnoea (or fatigue, palpitation or anginal pain). |
| Class IV – | Patients with cardiac disease resulting in inability to carry out physical activity without discomfort; symptoms of dyspnoea (or of angina) may be present even at rest; if any physical activity is undertaken, discomfort is increased. |

## Appendix 4: COVID-age calculation

[https://alama.org.uk/covid-19-medical-risk-assessment/](https://alama.org.uk/covid-19-medical-risk-assessment/) (last accessed September 2020).

The COVID-age calculation tool authored by the Association of Local Authority Medical Advisors (ALAMA), will be used to establish participant eligibility for the SPIKE1 trial.  

The tool was originally created to help assess an individual’s vulnerability to COVID-19 as part of an occupational health assessment of fitness for work. It is not intended for use in clinical treatment pathways. As new scientific evidence becomes available, its estimates of vulnerability may change. 

COVID-age helps assess an individual’s vulnerability to COVID-19. It is based on published evidence for the main identified risk factors. 

COVID-age summarises vulnerability for combinations of risk factors including age, sex and ethnicity and various health problems. It works by “translating” the risk associated with each factor into years which are added to (or subtracted from) an individual’s actual age.  This then gives a single overall measure of vulnerability. It can be used in people with no underlying medical conditions or multiple medical conditions. One measure combines all of an individual’s risk factors with their actual age.

COVID-age is translated into a risk level of low, moderate, high and very high. Participants who score moderate, high or very high, are eligible for SPIKE1.

There are times when the internet is not available or the ALAMA website goes down for maintenance. The risk tables can be downloaded as an Excel spreadsheet calculator in the link above. 

[^1]: The Medicines for Human Use (Clinical Trials) Regulations (S.I. 2004/1031) and any subsequent amendments to it.
[^2]: ICH Harmonised Guideline Integrated Addendum to ICH E6: Guideline for Good Clinical Practice E6(R2) Step 4 dated 09 November 2016
[^3]: WMA Declaration of Helsinki - Ethical Principles for Medical Research Involving Human Subjects adopted by the 18th WMA General Assembly, Helsinki, Finland, June 1964 and all subsequent amendments including Oct 2013.
[^4]: 1 The Medicines for Human Use (Clinical Trials) Regulations (S.I. 2004/1031) and any subsequent amendments to it. 2 ICH Harmonised Guideline Integrated Addendum to ICH E6: Guideline for Good Clinical Practice E6(R2) Step 4 dated 09 November 2016  3WMA Declaration of Helsinki - Ethical Principles for Medical Research Involving Human Subjects adopted by the 18th WMA General Assembly, Helsinki, Finland, June 1964 and all subsequent amendments including Oct 2013.
[^5]: All PK and plasma concentrations relate to GBPA, the active metabolite of camostat and not to the parent molecule. This due to rapid metabolism of camostat in vivo, however, the potency of both are considered to be equivalent.
[^6]: The PSRB acts as an independent body across the portfolio of studies at the CR UK Centre for Drug Development (CDD). The PSRB charter mandates that the members are independent from CR UK, CDD and can act as an independent body.
